# A spatiotemporal comparative analysis on tumor immune microenvironment characteristics between neoadjuvant chemotherapy and preoperative immunotherapy for ESCC

**DOI:** 10.1038/s41419-024-06986-y

**Published:** 2024-09-10

**Authors:** Zhengyang Zhou, Hongdian Zhang, Jian Du, Jiayu Yang, Wen Pan, Qiumo Zhang, Huiya Wang, Peng Tang, Yi Ba, Haiyang Zhang

**Affiliations:** 1 National Clinical Research Center for Cancer, Key Laboratory of Cancer Prevention and Therapy, Tianjin Medical University Cancer Institute and Hospital, Tianjin, 300202 China; 2 Department of Medical Oncology, Peking Union Medical College Hospital, Chinese Academy of Medical Sciences and Peking Union Medical College, No.1 Shuai Fu Yuan, Dongcheng District, Beijing, 100032 China; 3 Tianjin Institute of Coloproctology, Department of Colorectal Surgery, Tianjin Union Medical Center, Nankai University, Tianjin, 300121 China

**Keywords:** Cancer microenvironment, Cell death and immune response, Predictive markers

## Abstract

The average five-year survival rate for esophageal cancer, a common malignant tumor of the digestive system, is barely 20%. The majority of esophageal squamous cell carcinoma (ESCC) patients had already progressed to a locally advanced or even advanced stage at initial diagnosis, making routine surgery ineffective. Chemotherapy and immunotherapy are important neoadjuvant treatments for ESCC, however, it remains unknown how treatment will affect the immunological microenvironment, especially at the spatial level. Here, we presented the TME characters of ESCC from the temporal and spatial dimensions using scRNA-seq and ST, investigated the changes of immune cell clusters in the TME under neoadjuvant chemotherapy and preoperative immunotherapy, and explored the potential mechanisms. It was found that compared with chemotherapy, immunotherapy combined with chemotherapy increased the level of T cell proliferation, partially restored the function of exhausted T cells, induced the expansion of specific exhausted CD8 T cells, increased the production of dendritic cells (DCs), and supported the immune hot microenvironment of the tumor. We also found that CD52 and ID3 have potential as biomarkers of ESCC. Particularly, CD52 may be served as a predictor of the efficacy to screen the advantaged population of different regimens. Through multiple pathways, CAF2 and CAF5’s antigen-presenting role affected the other fibroblast clusters, resulting in malignant transformation. We analyzed the immune microenvironment differences between the two regimens to provide a more thorough description of the ESCC microenvironment profile and serve as a foundation for customized neoadjuvant treatment of ESCC.

## Introduction

Esophageal cancer has a very high incidence and mortality since it is a common malignant tumor of the digestive system [1]. China has a high prevalence of esophageal squamous cell carcinoma (ESCC), with new cases making over half of the annual global total [2]. Due to the fact that many early ESCC symptoms are not readily apparent and China has a low gastroscopy screening rate, the majority of patients can only receive a late diagnosis [3, 4]. Currently, surgery has a limited impact in treating advanced ESCC; instead, chemotherapy, immunotherapy, and radiotherapy are frequently needed [5]. Immunotherapy and chemotherapy-based ESCC clinical trials are currently ongoing and showing promising results [6, 7]. Since immunotherapy’s success in treating advanced ESCC, its function in adjuvant therapy and neoadjuvant therapy has also received considerable attention. However, the composition and changes of the immune microenvironment after ESCC treatment, the spatial distribution and interactions of various cell clusters have not been clearly demonstrated.

Tumor microenvironment (TME) has steadily grown in popularity as a study topic in recent years. Important TME constituents include immune cells, fibroblasts, adjacent vascular tissue, and extracellular matrix [8]. Tumor development, metastasis, and medication resistance are all significantly influenced by the ongoing interaction between tumor cells and TME [9]. Particularly significant is the role played by immune cells in the TME, which can either have anti- or pro-tumor actions. By examining gene expression in a single cell, single-cell RNA-sequencing (scRNA-seq) is a potent method for identifying cell variety [10]. ScRNA-seq allows for a more convenient exploration of tumor heterogeneity and its intricate relationship with the TME in the field of tumor research [11, 12]. Additionally, by restructuring the intricate relationships between immune cells, scRNA-seq may be able to uncover potential immunological response mechanisms [13]. Spatial transcriptomics (ST) can be used to overcome some of the shortcomings of scRNA-seq, such as the lack of spatial information about tissue samples. ST is a method that was first used in spatiotemporal mapping in 2016 and allows for the viewing and quantitative study of the transcriptome at a spatial resolution in tissue slices [14, 15]. Furthermore, ST can examine the intrinsic heterogeneity of tumor tissues and provide an explanation for how TME changes in space [16]. Both the identification of novel tumor biomarkers and the giving of therapeutically beneficial advice for diagnosis and prognosis have benefited greatly from ST [17].

This study used tumor tissues and matched adjacent normal tissues from ESCC patients who had undergone neoadjuvant chemotherapy and immunotherapy before surgery to examine the changes in cell clusters and cell components of the tumor immune microenvironment under different regimens. When immunotherapy was added into the regimen, the amount of T cell proliferation increased, the inactivation process of exhausted T cells was partially reversed, and tumor development was pushed in the direction of the immune heat microenvironment. It was found that CD52 may be the potential biomarker for identifying privileged groups. Meanwhile, the interconnections of various cell clusters and their spatial linkages in the tumor site were examined. A more detailed ESCC profile was developed to serve as the basis for individualized ESCC neoadjuvant therapy, and patients with ESCC may benefit more from different regimens in the future.

## Results

### Single cell transcriptome map reveals tumor microenvironment characters of ESCC patients treated with two neoadjuvant regimens

We obtained tumor tissues (CA group) and adjacent normal tissues (NC group) from three patients receiving preoperative neoadjuvant chemotherapy and three patients receiving preoperative combination immunotherapy in order to map the single-cell ESCC and reveal the difference in TME between neoadjuvant chemotherapy (chemo-group) or neoadjuvant immunotherapy combined chemotherapy (immuno-group). These six individuals had ESCC as their pathology diagnosis, and Table 1 displays their clinical details. Additionally, we also followed up some ESCC patients who underwent surgery in our hospital from December 2019 to June 2022, and the baseline statistics were presented in Table 2.

**Table 1:** Clinical information of patients.

Number	Gender	Age range	Pathological diagnosis	Histological grade	Treatment regimens
chemo_1	Male	66–70	ESCC	moderately differentiation	Nedaplatin+Paclitaxel
chemo_2	Male	66–70	ESCC	poorly differentiation	Cisplatin+Paclitaxel
chemo_3	Male	60–65	ESCC	moderately differentiation	Cisplatin+Paclitaxel
immuno_1	Female	71–75	ESCC	moderately differentiation	Nedaplatin+Paclitaxel+Sintilimab
immuno_2	Male	60–65	ESCC	poorly differentiation	Nedaplatin+Paclitaxel+Sintilimab
immuno_3	Male	60–65	ESCC	moderately differentiation	Nedaplatin+Paclitaxel+Sintilimab

All 6 patients received neoadjuvant chemotherapy or neoadjuvant immunotherapy combined with chemotherapy. These patients had not previously undergone any other surgery or treatment. The pathological type was ESCC. Histological grade was moderate or low differentiation. The neoadjuvant chemotherapy group was treated with nedaplatin or cisplatin combined with paclitaxel. Neoadjuvant immunotherapy combined with chemotherapy was treated with nedaplatin, paclitaxel and sintilimab.

**Table 2:** Baseline statistics of clinical data of follow-up ESCC patients.

	Neoadjuvant chemotherapy (chemo group)	Neoadjuvant immunotherapy combined chemotherapy (immuno group)	*P-*value
Number, *N*	62	102	
Gender, *N* (%)			0.285
Male	51 (36.2%)	90 (63.8%)	
Female	11 (47.8%)	12 (52.2%)	
Age, *N* (%)			0.126
> 60	38 (43.2%)	50 (56.8%)	
≤60	24 (31.6%)	52 (68.4%)	
Weight, *N* (%)			0.437
> 70	7 (38.9%)	11 (61.1%)	
≤70	16 (29.1%)	39 (70.9%)	
missing	39 (42.9%)	52 (57.1%)	
T stage, *N* (%)			0.138
T3	43 (44.3%)	54 (55.7%)	
T2	1 (16.7%)	5 (83.3%)	
T1	18 (31%)	40 (69%)	
missing	0	3 (100%)	
N stage, *N* (%)			0.827
N0	21 (34.4%)	40 (65.6%)	
N1	21 (42.9%)	28 (57.1%)	
N3	8 (40%)	12 (60%)	
N2	12 (36.4%)	21 (63.6%)	
Missing	0	1 (100%)	
Differentiated degree, *N* (%)			
poorly differentiation	4 (33.3%)	8 (66.7%)	
medium-low differentiation	16 (40.7%)	29 (59.3%)	
medium differentiation	35 (35.6%)	51 (64.4%)	
high differentiation	1 (33.3%)	2 (66.7%)	
missing	6 (33.3%)	12 (66.7%)	
Pathological stage, *N* (%)			0.769
I	8 (39.1%)	19 (60.9%)	
II	18 (29.6%)	28 (70.4%)	
III	28 (40.6%)	41 (59.4%)	
IV	8 (42.1%)	11 (57.9%)	
missing	0	3 (100%)	
Recurrence and metastasis, *N* (%)			0.208
no	60 (39.5%)	92 (60.5%)	
yes	2 (16.7%)	10 (83.3%)	
Survival, *N* (%)			0.890
no	17 (34%)	33 (66%)	
yes	25 (35.2%)	46 (64.8%)	
missing	20 (46.5%)	23 (53.5%)	

Follow-up baseline statistics of ESCC patients who underwent surgical treatment between December 2019 and June 2022 summarized their gender, age, differentiated degree, pathological stage, survival, and more.

**Fig. 1:**
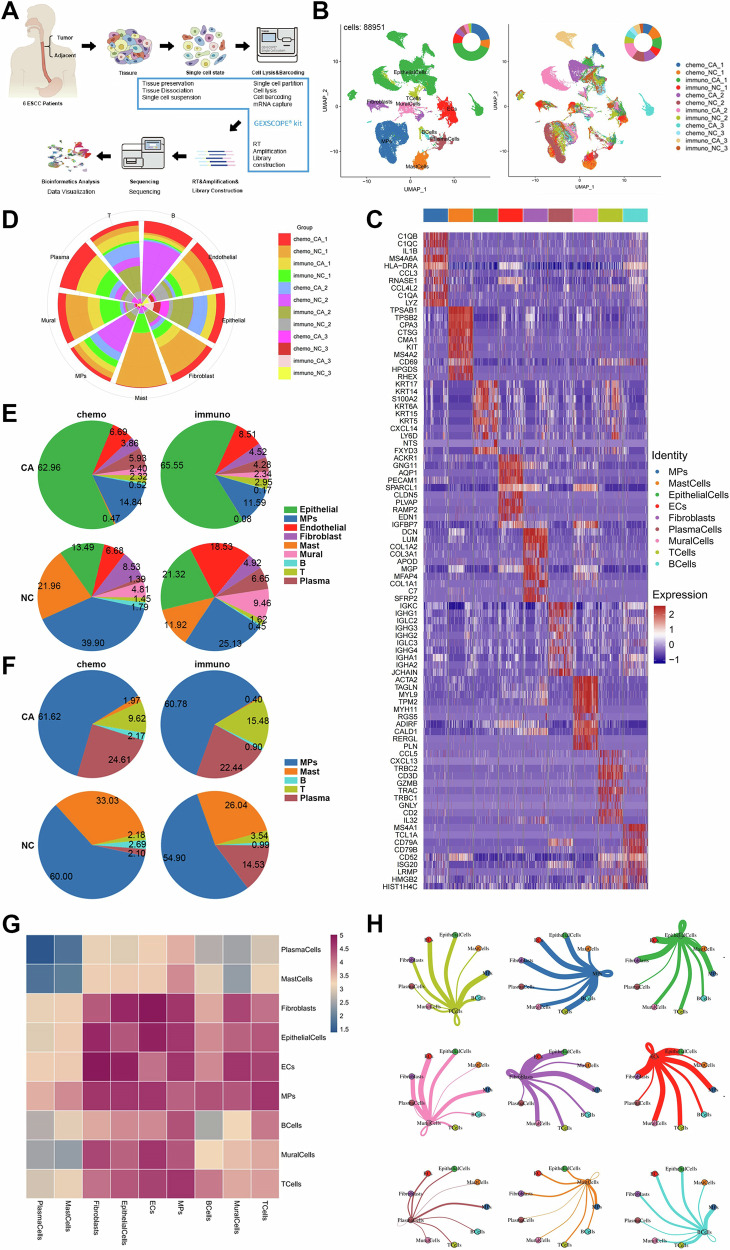
Single cell transcriptome map and major cell types in ESCC tumor microenvironment. **A** Single cell sequencing process. **B** Cell types and UMAP of all samples. **C** Heat maps of typical marker gene expression by cell types. **D** The proportional contribution of tissue samples to each cell type. **E** Proportion of each cell type in the four groups. **F** Proportion of immune cell components in four groups. The interactions between cell types shown in the form of (**G**) heat map and (**H**) shell map. *P* < 0.05. *P < 0.05; **P < 0.01; ***P < 0.001.

First, scRNA-seq was carried out on all of the tissue samples’ isolated cells (Fig. 1A). A total of 88951 cells (2876–12741 each sample) were kept for further investigation after low-quality cells were eliminated. Unsupervised clustering was used to assess the cell composition profile in the microenvironment of ESCC tumor tissue as well as adjacent normal tissue utilizing sequencing data to systematically investigate these cell types. We did not batch process the data to avoid improper removal of tumor heterogeneity. Nine major cell types, including epithelial cells, T cells, mononuclear phagocytes (MPs), fibroblasts, endothelial cells (ECs), mural cells, plasma cells, mast cells, and B cells, were successfully recognized after being annotated using their canonical markers (Fig. 1B). The annotation was consistent with how the nine different cell types’ usual markers were expressed. (Fig. 1C). A significant cell types were contributed to by each patient sample (Fig. 1D). For example, chemo_2_NC was dominant in B cells and mononuclear phagocytes, immuno_2_CA was dominant in T cells and epithelial cells. Based on the aforementioned results, we presented and described the complete ESCC transcriptome map, laying the groundwork for an in-depth investigation of the features of the ESCC microenvironment.

We observed significant heterogeneity in the cell makeup of various samples by comparing the percentage of each cell type in all tissue samples (Supplementary Fig. 1A). Next, the epithelial cells with the highest percentage in the majority of tissue samples were chosen for examination. Basal cells, secretory cells, keratinized esophageal squamous cells, and cancer cells were identified as the four main cell clusters of these cells (Supplementary Fig. 1B). Based on heterogeneity, we defined cancer cells as clusters of epithelial cells with high heterogeneity. Between clusters of epithelial cells, we discovered significant differences in gene expression (Supplementary Fig. 1C). The identity of these cancer cells was further validated by CNV analysis, including amplification and deletion (Supplementary Figure 1D). Additionally, Supplementary Fig. 1E illustrated the robust activity and proliferative ability of cancer cells. These findings validate our description of cancer cells by revealing that malignant cells in epithelial cells are ESCC.

We integrated the scRNA-Seq data of all tissue samples (divided into chemo_CA, chemo_NC, immuno_CA, and immuno_NC group) to examine the differences in various cellular components as well as the impact of two regimens on TME of ESCC. In the CA group, epithelial cells made up the majority of cell subsets, whereas MPs made up the majority of cell types in the NC group (Fig. 1E). The immuno-group had a higher percentage of T cells and a lower percentage of MPs when compared to the chemo-group. Then, the whole cell subsets were divided into immune cell components and non-immune cell components. In terms of immunological components, we found that while the proportion of plasma cells and T cells significantly increased in the CA group compared to the NC group, the mast cell fraction significantly decreased (Fig. 1F). Furthermore, we observed a considerable increase in the proportion of T cells, indicating that the administration of Sintilimab may cause peripheral T cell activation and subsequent recruitment into tumors. According to a report, ESCC was one of the cancer kinds with more MPs and T cells infiltrating but less B cells entering when compared to other more common malignancies [18]. This is compatible with our observations.

We explored the interactions among cell types in ESCC, as shown in heat maps and shell maps (Fig. 1G, H). T cells interacted most strongly with MPs, whereas fibroblasts interacted most strongly with MPs, mural cells, endothelial cells, and epithelial cells. In addition, MPs communicated with B cells, ECs, and epithelial cells. The top 20 interaction partners are also included in Table 3.

**Table 3:** The top 20 interaction pairs between cell types.

Ligand cell	Receptor cell	Ligand gene	Receptor gene	Secreted
Epithelial cells	T cells	TNFSF9	TNFRSF9	√
Fibroblasts	Fibroblasts	DPP4	CCL11	√
Mast cells	Fibroblasts	ADCYAP1	DPP4	√
Epithelial cells	B Cells	TNFSF9	ADGRG5	√
T cells	Epithelial cells	XCL1	ADGRV1	√
ECs	Epithelial cells	PDGFB	ADGRV1	√
Mural cells	ECs	CCL8	ACKR1	√
ECs	Fibroblasts	PDGFB	PDGFR	√
B cells	T cells	TNFRSF13B	CD70	√
Epithelial cells|	Plasma cells	CDH1	aEb7	×
ECs	Fibroblasts	PDGFB	PDGFRA	√
Epithelial Cells	ECs	COL9A3	a10b1	√
Epithelial cells	ECs	EFNA3	EPHA4	×
Fibroblasts	ECs	COL16A1	a10b1	√
Epithelial cells	ECs	EFNA4	EPHA4	√
Fibroblasts	ECs	COL10A1	a10b1	√
Fibroblasts	ECs	VEGFD	KDR	√
Fibroblasts	ECs	COL4A4	a10b1	√
Epithelial cells	Mast cells	PLXNB1	SEMA4D	√

Interaction pairs between cell types in ESCC was explored and the top 20 interaction pairs was displayed. Ligand cells, receptor cells, ligand genes and receptor genes are labeled separately. If the interaction is in the form of secretion, it is “√“, otherwise it is “×“.

### Clustering and subtype analysis of T cells in ESCC tumors after neoadjuvant chemotherapy or preoperative immunotherapy

**Fig. 2:**
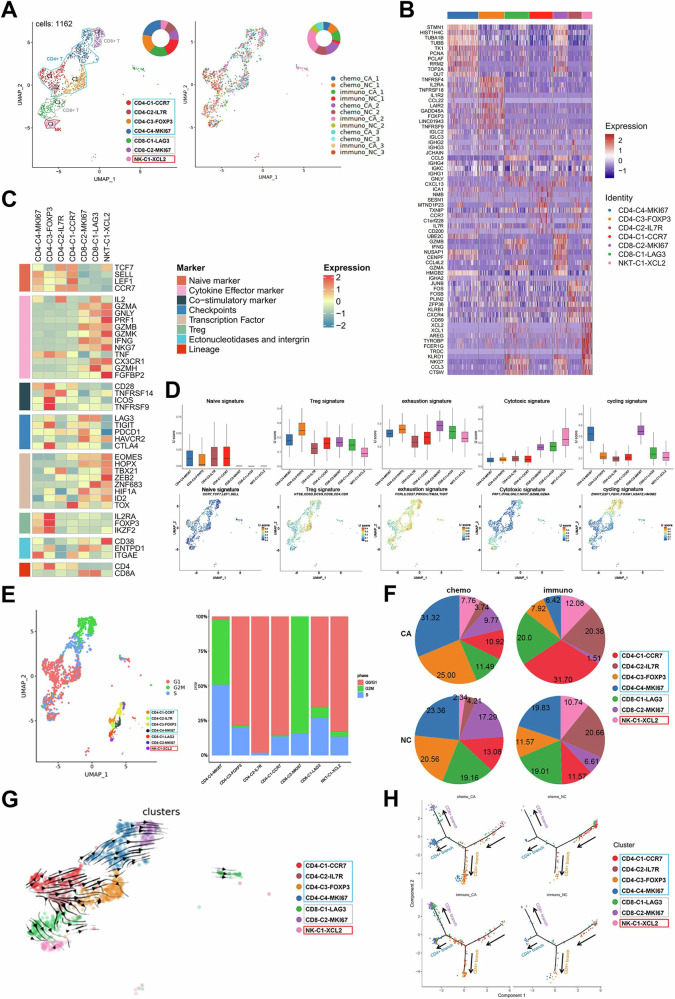
Clustering and subtype analysis of T cells in ESCC after neoadjuvant therapies. **A** UMAP of T cell clusters and all tissue samples. **B** Heat map of typical marker gene expression in T cell clusters **C** Expression levels of classic markers in T cell clusters. **D** Functional characteristics of T cell clusters scored in box type and UMAP. **E** Cell cycle UMAP of T cell clusters and cell cycle ratio map of four groups. **F** Proportion of T cells clusters in four groups. **G** RNA rate diffusion of T cell clusters. **H** Pseudotemporal analysis of T cell clusters in four groups. *P* < 0.05. *P < 0.05; **P < 0.01; ***P < 0.001.

We concentrated on investigating the immune microenvironment modifications associated with ESCC and any potential regulatory pathways or mechanisms. First, we analyzed T cells, the main lymphocyte population and important cytotoxic immune cells in the tumor immune microenvironment. Out of a total of 1162 cells, we performed unsupervised clustering of T cells from all samples and found four CD4 clusters, two CD8 clusters, and one NK cells cluster (Fig. 2A). Figure 2B depicted the top differentially expressed genes (DEGs) for each cluster. The known functional markers were used to suggest CD4 and CD8 T cells, including naïve, exhausted T cells, and Tregss, as well as marker genes for cytokines, co-stimulators, and immune checkpoints (Fig. 2C). CD4-C1-CCR7 and CD4-C2-IL7R carried the most classic naïve signature CCR7 and IL7R. TCF7, LEF1, and SELL were also expressed at high levels. FOXP3, IL2RA, CTLA4, and IKZF2 were highly expressed Treg characteristic markers on CD4-C3-FOXP3. The co-stimulatory markers, including TNFRSF9, ICOS, and CD28, were likewise strongly expressed in this cluster. CD4-C4-MKI67 were characterized by both naïve and Tregs, with high expression of SELL, LEF1, and ITGAE. It is interesting to note that CD4-C4-MKI67 had significant levels of MKI67 expression, indicating great proliferative potential. Two CD8 T clusters had high levels of the cytokines IFNG, GZMA, and NKG7 as well as the checkpoint molecule genes LAG3, TIGIT, PDCD1, HAVCR2, and CLTA4, which were indicative of the phenotypes of exhausted cells. Based on a study [19], IFNG and GZMB were two cytotoxic signatures that are strongly expressed in exhausted CD8 T cells in addition to TNF and IL2. This expression was also shown in CD8-C1-LAG3 and CD8-C2-MKI67. The cell cycle-reflecting genes MKI67, CDKN3, and CCNB1 were highly expressed in CD8-C2-MKI67. These two clusters, however, also displayed elevated CXCL13 expression, demonstrating that they were tumor-associated infiltrating CD8 T cells. NKT-C1-XCL2 was the sole NK cell collection. In addition to highly expressed cytotoxic signatures like NKG7, GNLY, and GZMB, as well as transcription factors like ZEB2, TBX21, and EOMES, this cluster also contained highly expressed NK cell markers including KLRD1, FCGR3A, and KLRC1.

For aiding with identification, various public signatures were applied to T cell clusters, and scores were computed (Fig. 2D). It was observed that the exhaustion scores of CD8-C2-MKI67, CD8-C1-LAG3, and CD4-C4-MKI67 were greater. It is interesting to note that compared to the other groups, CD4-C4-MKI67 and CD8-C2-MKI67 both had much higher proliferation scores. In terms of cell cycle proportion, these two clusters likewise showed high levels of proliferation (Fig. 2E). This is in line with a recent study that found the exhausted T cells to be a significant immune cell compartment that proliferates in malignancies [20]. It was also discovered that CD8 T cells greatly outperformed CD4 T cells in terms of cytotoxicity. According to a study, CD8 T cells that were highly expressed in cytotoxic genes also exhibited high levels of the NK cell receptor markers KLRD1, KLRC1, and KLRC2. In CD8-C1-LAG3 and CD8-C2-MKI67, the same pattern was identified.

To confirm these clusters’ possible roles, KEGG functional enrichment analysis was carried out (Supplementary Fig. 2A). Two immune checkpoint markers CTLA4 and PDCD1 were detected to explore their expression in each T cell clusters (Supplementary Fig. 2B). Except for NKT-C1-XCL2, all clusters that we examined expressed CTLA4, but CD4-C3-FOXP3 had the highest level, suggesting that CTLA4 was a key player in the immunosuppression of Tregs. Evidently, PDCD1 participated in the process of T cell exhaustion because it was only expressed in CD8-C1-LAG3. It was reported that PD-1 was significantly expressed in exhausted CD8 T cells, and in contrast to CTLA4 blocking, the effect of PD-1 blocking may be concentrated in remaining tumor T cells [21].

The changes in the proportion of T cells were next examined. In Supplementary Fig. 2C, the clusters from all tissue samples were displayed. After the combination, we found that the NC group had a higher percentage of naïve T cells (CD4-C1-CCR7 and CD4-C2-IL7R) (Fig. 2F). The immuno-group had a larger percentage of naïve T cells than the chemo-group, indicating that the administration of immunological medicines boosted T cell proliferation. Additionally, we found that CD8-C2-MKI67, a cluster with high PDCD1 expression, was higher in the CA group compared to the NC group, particularly in the immuno-group compared to the chemo-group. This finding suggests that Sintilimab may reverse T cell exhaustion and induce specific tumor infiltrating exhausted CD8 T cell. The difference between the proportions of Tregs (CD4-C3-FOXP3) in the immuno- and chemo-groups could be explained by Sintilimab’s ability to prevent Treg growth. The proportion of NK cells in the CA group reduced as compared to the NC group, indicating that there weren’t enough NK cells and that their function was compromised in ESCC. Considering to the above findings, the clinical administration of the PD1 inhibitor Sintilimab reversed the exhausted T cells, partially restored T cell function, and enhanced the tumor’s immune hot environment.

The potential developmental pathways of cell conversion were built using Monocle 2 to study the cell transitions (Supplementary Fig. 2D). CD4-C3-FOXP3, CD4-C4-MKI67, and CD8-C2-MKI67 were found in the medium and end stages of differentiation, whereas CD4-C1-CCR7, CD4-C2-IL7R, and NKT-C1-XCL2 were largely dispersed in the early stage of differentiation. Using the RNA rate diffusion diagram in conjunction, we found that some CD4-C2-IL7R differentiated into CD8-C1-LAG3 and some CD4-C1-CCR7 differentiated into CD4-C3-FOXP3 (Fig. 2G). In the middle and end stages, CD4-C3-FOXP3 differentiated into CD4-C4-MKI67, and the latter differentiated even more into CD8-C2-MKI67. Moreover, it was discovered that IGHA2, IGKC, IGLC2, JCHAIN, and other genes were strongly expressed at the end of development, whereas FABP4, FABP5, LY6D, and other genes were significantly expressed in the beginning of development (Supplementary Figure 2E). Meanwhile, we observed that naïve T cells in the immuno-group were stacked in front of the “fork in the road” leading to the fate of CD4 or CD8 T cells, and the majority of them ended up in exhausted CD8 T cells, but naïve T cells in the chemo-group largely ended up in CD4-C3-FOXP3 and CD4-C4-MKI67 (Fig. 2H). The aforementioned findings imply that T cell clusters are not entirely independent but may be in a broad state of mutual change, and studies that support our hypothesis can be found 75. We also analyzed transcription factors (TFs) and found significant differences in regulatory networks between chemo_NC group and immuno_NC group (Supplementary Figure 2F).

### Construction of CD4 T cell risk score model

**Fig. 3:**
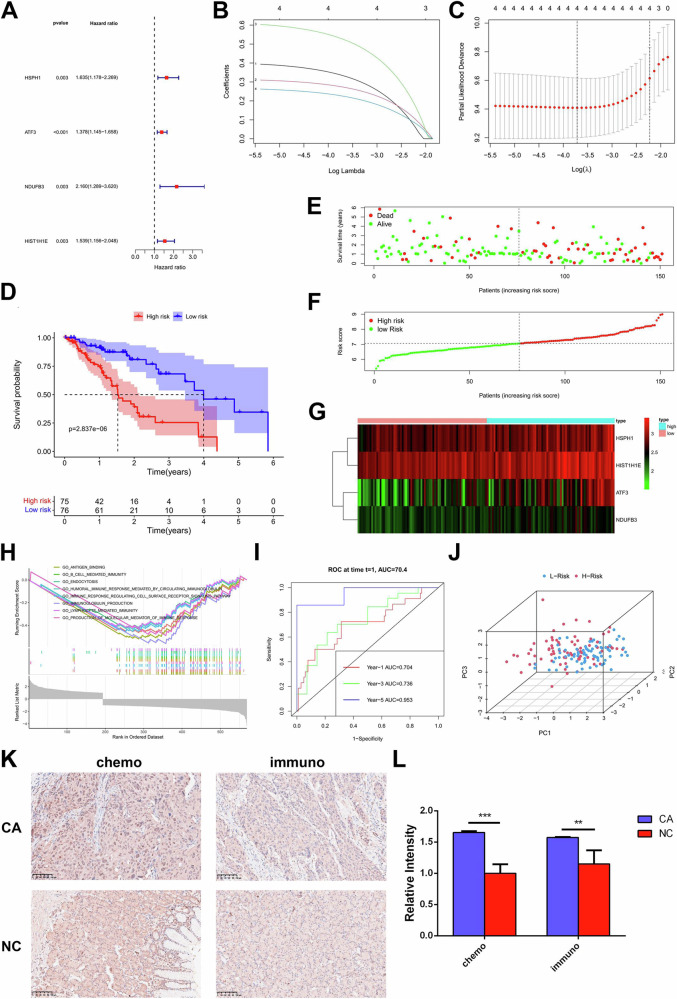
Construction of CD4 T cell risk score model. **A** CD4 T cell unifactor Cox analysis of forest map. **B**, **C** LASSO regression analysis diagram. **D** KM survival curves of high-risk group and low-risk group. **E** Survival map of ESCC patients in TCGA. **F** Risk score map of ESCC patients in TCGA. **G** Heat maps of expression levels of four T cell marker genes in high-risk and low-risk groups. **H** GSEA pathway enrichment analysis diagram. **I** 1/3/5 year ROC curve of the risk scoring model. **J** PCA diagram of risk assessment model. **K**, **L** IHC analysis of the paraffin-embedded ESCC tissues using a HSPH1 antibody and the corresponding quantification. *P* < 0.05. *P < 0.05, **P < 0.01; ***P < 0.001.

T cells in the TME are crucial in either promoting or inhibiting the growth of tumors. It was reported that CD4 T cells and the prognosis of tumor patients were associated. Then, in hopes to facilitate clinical treatment, we constructed a risk score model using the DEGs of CD4 T cells. First, using the ESCC transcriptome data from TCGA, we developed the model and then screened the 371 CD4 T cell marker genes for genes with |LogFC | > 0.5, *P* < 0.01. By using univariate Cox analysis and LASSO regression analysis, four genes—HSPH1, ATF3, NDUFB3, and HIST1H1E—were identified, and the risk score for each patient was determined (Fig. 3A–C).

We split the ESCC patients in the TCGA database into low-risk and high-risk groups based on their median risk scores, and the high-risk group’s overall survival time was considerably shorter than that of the low-risk group (Fig. 3D). Figure 3E, F displayed the risk and survival scores for each patient with ESCC. The expression levels of four genes in each patient were depicted on the gene heat map (Fig. 3G). The DEGs between the two groups were associated with immunoregulatory pathways like antigen binding, humoral immune response mediated by circulating immunoglobulin, immune response regulating cell surface receptor signaling pathways, and lymphocyte-mediated immunity, according to GSEA enrichment analysis (Fig. 3H). To calculate the risk scoring model’s accuracy, we employed the time ROC curve. Patients with ESCC had AUC values of 0.704, 0.736, and 0.953 at 1, 3, and 5 years, respectively (Fig. 3I). Four prognostic genes’ expression levels significantly distinguished patients in the low-risk group, according to PCA analysis (Fig. 3J). The information shown above clearly demonstrates that our CD4 T cell risk score model’s prognosis is reliable. We also conducted IHC on HSPH1 (Fig. 3K). According to the findings, the CA group had higher levels of HSPH1 expression than the NC group (Fig. 3L). The expression level of HSPH1 did not significantly differ between the two treatments.

### ID3 and CD52 serve as markers to predict the efficacy of two neoadjuvant treatment regimens for ESCC

**Fig. 4:**
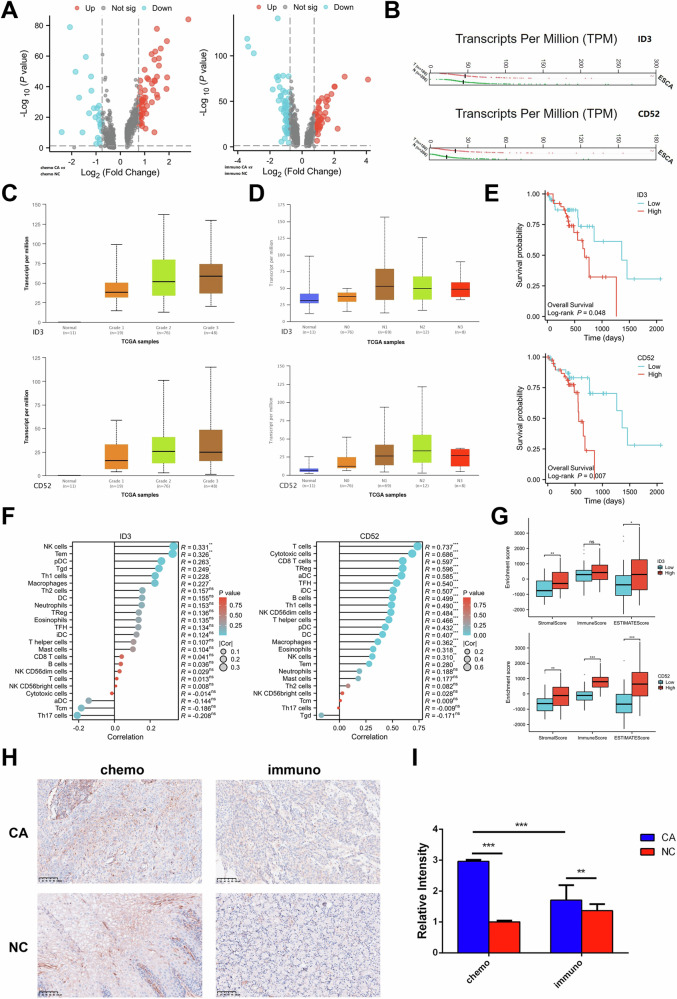
ID3 and CD52 can be used as markers to predict the efficacy of neoadjuvant therapies for ESCC. **A** Differential gene volcano map between tumor group and adjacent normal tissue group under two regimens. **B** Expression levels of ID3 and CD52 in ESCC tumor tissue and normal tissue. **C** The correlation between the expression level of ID and CD52 and the differentiation degree of ESCC shown in the histogram. **D** ID3, CD52 expression level and ESCC metastasis correlation histogram. **E** KM survival curves of ID3 and CD52. **F** Lollipop diagram of immune cell infiltration correlation of ID3 and CD52 in ESCC. **G** Bar chart for the ESTIMATE score of ID3 and CD52 in ESCC. **H**, **I** IHC analysis of the paraffin-embedded ESCC tissues using a CD52 antibody and the corresponding quantification. *P* < 0.05. *, *P* < 0.05; **P < 0.01; ***P < 0.001.

We compared T cells between the chemo-group and the immuno-group to investigate DEGs under two regimens and their possible function in TME. First, the DEGs between the immuno_CA group and the immuno_NC group, as well as between the chemo_CA group and the chemo_NC group, were examined (Fig. 4A). When up-regulated and down-regulated DEGs in the chemo_CA group and immuno_CA group were intersected, a total of 272 DEGs and 178 DEGs were found in the up-regulated group, respectively. ID3 and CD52 are two of these DEGs that have captured our interest. ID3 is involved in various cellular processes, including apoptosis, angiogenesis, and tumor transformation [22]. The LEF1/ID3/HRAS axis has been linked to several research’ findings that it can encourage the emergence and progression of ESCC [23]. On the surface of mature lymphocytes, neutrophils, macrophages, DCs, and other immune cells, CD52 is abundantly expressed [24, 25]. In addition, it has been noted that CD52 is shed from the cell surface and remains in the blood; these soluble CD52 molecules may be employed as biomarkers for biological detection [24].

ID3 and CD52 expressions were found in both normal and malignant tissues. In the GEPIA database, it was demonstrated that tumor tissues had higher levels of ID3 and CD52 expression than normal tissues (Fig. 4B). We investigated whether the expression of ID3 and CD52 was related to cancer features using the UALCAN database. The expression levels of ID3 and CD52 gradually increase as the degree of ESCC differentiation declines, suggesting that the degree and progression of ESCC differentiation may be assessed by the expression levels of ID3 or CD52 (Fig. 4C). Additionally, we found that ESCC patients with lymph node metastases frequently had higher ID3 and CD52 expressions than ESCC patients without lymph node metastasis (Fig. 4D). The TCGA database showed that high levels of ID3 and CD52 expression were linked to a poor prognosis in ESCC patients, indicating that these proteins may be oncogenes in ESCC and have the ability to act as prognostic indicators (Fig. 4E).

The roles of these two genes in the immunological microenvironment of the ESCC were then investigated in connection to immune cells. In breast invasive carcinoma, adrenal cortical carcinoma, adrenal carcinoma, and a variety of malignant tumors, including ESCC, we discovered that ID3 was positively correlated with many immune cells (CD8 T cells, NK cells, DCs, macrophages, etc.), whereas CD52 was significantly positively correlated with all immune cells in almost all malignancies, and ESCC was no exception (Supplementary Fig. 3A). The link between the expression of ID3 and CD52 in ESCC and the infiltration of different immune cells was confirmed using the TIMER database (Fig. 4F). We noticed that the presence of NK cells, effector memory T cells, plasmacytoid DCs, type 1 helper T cells, and macrophages was positively linked with ID3. T cells, cytotoxic cells, CD8 T cells, Tregs, DCs, macrophages, NK cells, and other immune cell infiltration were all positively linked with CD52. Activated T cells, central memory T cells, effector memory T cells, Tregs, macrophages, and other immune cell components were all strongly correlated with CD52, according to the TISIDB database, which further demonstrated that CD52 was likely involved in the immune regulation process in the TME of ESCC (Supplementary Fig. 3B). We assessed the level of immune infiltration of ID3 and CD52 in ESCC using the ESTIMATE method (Fig. 3G). It was found that immune infiltration and matrix component levels were much higher when CD52 was abundantly expressed, indicating that CD52 increased the invasiveness of ESCC. The existence of matrix in tumor tissue and higher tumor purity were solely indicated by the high expression of ID3, which did not imply a connection with the aggressiveness of ESCC.

To determine the expression of CD52 in ESCC tissue samples, IHC were performed (Fig. 3H). Regardless of the treatment, the CA group’s CD52 expression level was higher than that of the NC group (Fig. 3I). In the immuno-group, CD52 expression was lower than it was in the chemo-group, suggesting that Sintilimab inhibited CD52 expression. These findings imply that ID3 and CD52, particularly CD52, which is strongly associated to the tumor immune milieu, are favorably correlated with immune cells and immune infiltration in ESCC. Both of them have the potential to be prognostic indicators of ESCC and may function as oncogenes in the development of ESCC.

### Clustering and subtype analysis of MPs

**Fig. 5:**
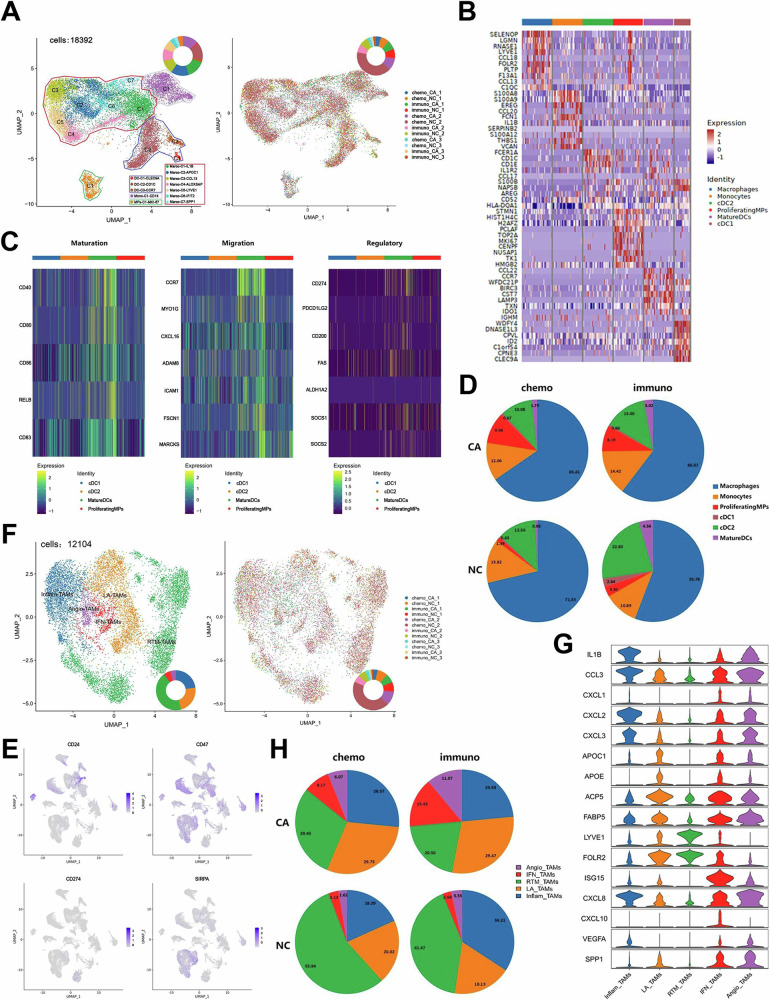
Clustering and subtype analysis of MPs. **A U**MAP of MPs clusters and all tissue samples. **B** Heat map of typical marker gene expression in each cluster of MPs. **C** Heat map of mean expression levels of mature, migratory, and regulatory genes in DCs. **D** Proportion of MPs clusters in four groups. **E** UMAP of TAMs clusters and all tissue samples. **F** UMAP of TAMs clusters and all tissue samples. **G** Violin map of typical differential genes in each cluster of TAMs. **H** Proportion of TAMs clusters in four groups. *P* < 0.05. *P < 0.05; **P < 0.01; ***P < 0.001.

We then examined the MPs found in immunological cells. Out of a total of 18392 cells, we unsupervisedly clustered MPs from all samples and found four clusters. Monocytes, macrophages, DCs, and a distinct cluster of MPs (MPs-C1-MKI67) with classical proliferative marker genes made up this group (Fig. 5A). Figure 5B displayed each cluster’s top DEGs. The macrophage, which made up the majority of these cells and accounts for 65.8% of MPs (Supplementary Fig. 4A), had seven distinct cell clusters. We distinguished each cell cluster based on the expression of particular genes. For example, the three DC clusters were categorized as mature dendritic cells (mDCs), type II classical dendritic cells (cDC2), and type I classical dendritic cells (cDC1). MPs that express MKI67 and TOP2A strongly were categorized as proliferating MPs (proMPs). The elevated expression of CD83, CCR7, and LAMP3 in mDCs was consistent with research on osteosarcoma. Tumor-associated CD83 + CCR7 + LAMP3+ DCs were found to selectively express in tumor tissue [26]. The mDCs had active state, indicating that this cluster was a mature regulatory DCs, and were much more mature than cDC1 and cDC2 in terms of migration and regulatory abilities (Fig. 5C). A variety of invading T cells may be recruited by mDCs, which selectively expressed CCR7, CCL17, and CCL22 (Supplementary Figure 4B). In Supplementary Figure 4C, the makeup of each cluster in all tissue samples was presented. Comparing the immuno- and chemo-groups, it was found that the immuno-group had a reduced percentage of macrophages (Fig. 5D). Compared with the NC group, the CA group had a larger percentage of proMPs. The proportion of DCs was larger in the immuno-group compared to the chemo-group, which may be explained by the fact that immunotherapy caused the tumor’s immune system to become more active, and that more DCs were needed to cause a CD4 T cell response.

Monocytes and cDC2 were found in practically every stage, macrophages were more prevalent in the early and end stages of differentiation, and proMPs and mDCs were concentrated at the end stages of differentiation, according to the pseudo-time analysis (Supplementary Fig. 4D). The MP clusters in the CA group were more heavily concentrated in the middle and end stages of differentiation when compared to the NC group. Between the chemo- and immuno-groups, no discernible variations in differentiation were revealed. Furthermore, it was found that LILRB5, CD99, and other genes were strongly expressed at the conclusion of development, while HSP90AA1, SPP1, and other genes were substantially expressed at the beginning of the development trajectory (Supplementary Fig. 4E).

Numerous studies on TAMs have been published recently, which reveal a wide range of TME characteristics. Depending on their functional traits and the quantity of inflammatory substances they secrete, macrophages can be classified as M1 macrophages or M2 macrophages. To ascertain the phenotypes of seven clusters, we utilized the public signatures of these two macrophage types (Supplementary Fig. 4F). It was observed that macrophage clusters 1, 6, and 7 had higher M1 phenotype scores than other clusters, whereas macrophage clusters 2, 3, and 5 had higher M2 phenotype scores than other subgroups. In contrast, the M2 phenotype score of all clusters was higher overall.

Based on a study, tumor cells overexpressed CD47 and PD-L1 (CD274) to evade immune cell clearance [27]. Combining with CD24 to deliver the “do not eat me” signal, CD47, an immune checkpoint receptor that is frequently dysregulated in malignant tumors, inhibits macrophage-mediated phagocytosis. We found that CD24 and CD47 expression in macrophages was much stronger than CD274 expression, indicating that macrophage-mediated immunological escape rather than T cell-mediated immune escape was implicated in ESCC (Fig. 5E).

We repeated unsupervised macrophage clustering and found five clusters in a total of 12104 cells by defining the tumor associated tumors (TAMs) cluster gene sets according to various functions (Fig. 5F). They are resident-tissue macrophage-like TAMs (RTM-TAMs), lipid-associated TAMs (LA-TAMs), interferon-primed (IFN-TAMs), inflammatory cytokine-enriched TAMs (Inflam-TAMs), and proangiogenic TAMs (Angio-TAMs). Although the expression of the typical markers for these five clusters was consistent with the annotation, similar circumstances in the expression of these DEGs suggested that the clusters might be in a state of mutual change or transition (Fig. 5G).

Although the proportion of Inflam-TAMs was lower in the immuno_CA group compared to the immuno_NC group, it was higher in the chemo_CA group than the chemo_NC group, indicating that the use of immunotherapy may lessen the likelihood of an inflammatory response (Fig. 5H). Compared with the NC group, the proportion of RTM-TAMs in the CA group was less, indicating that the cluster was indeed enriched in adjacent normal tissues as reported in the study [28]. In comparison to the chemo-group, the immune group had fewer RTM-TAMs. LA-TAMs had a role in M2 macrophage function in TME, which may have an inhibitory influence on immunological response. The proportion of LA-TAMs in the CA group was considerably higher than that in the NC group.

### Analysis of fibroblast clusters and CAF in ESCC after two neoadjuvant treatment regimens

**Fig. 6:**
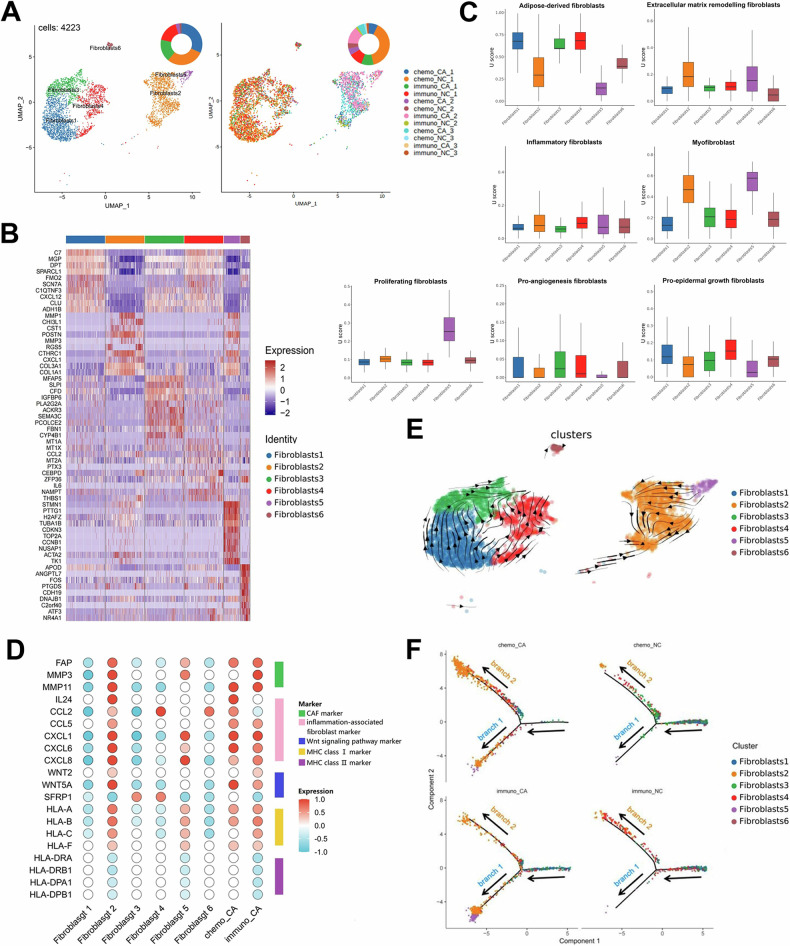
Analysis of fibroblast clusters and CAF in ESCC following neoadjuvant therapies. **A** UMAP of fibroblast clusters and all tissue samples. **B** Heat map of typical marker gene expression in each cluster of fibroblasts. **C** Functional characteristics of fibroblast clusters scored in box type. **D** Classical marker scores of each cluster of fibroblasts. **E** RNA rate diffusion of fibroblast clusters. **F** Pseudotemporal analysis of fibroblast clusters in four groups. *P* < 0.05. *P < 0.05; **P < 0.01; ***P < 0.001.

The ESCC matrix components were then investigated. First, we used unsupervised clustering to find six clusters out of a total of 4223 fibroblasts from all samples (Fig. 6A). Fibroblast clusters 1 and 2 were the most prevalent among them, accounting around 30% of all fibroblasts. Each cluster’s top DEGs were illustrated in Fig. 6B. Obviously, the chemo_NC group contributed more cells in fibroblast cluster 1, 3, and 4, while the CA group contributed more cells in fibroblast cluster 2 and 5 (Supplementary Fig. 5A). We noticed that the proportion of fibroblast cluster 2 and 5 in the CA group was higher than that in the NC group (Supplementary Fig. 5B). In the CA group, fibroblast clusters 1, 3, and 4 accounted for a smaller percentage. These findings indicate that fibroblast clusters 2 and 5 might be cancer-associated fibroblasts (CAFs). To identify the fibroblast clusters, various public signatures were applied, and scores were computed (Fig. 6C). We hypothesized that fibroblast clusters 2 and 5 in the TME were CAFs after it was shown that they scored highly on extracellular matrix remodeling fibroblasts, myofibroblasts, and proliferating fibroblasts.

Then, utilizing the known functional markers, such as the CAF markers, inflammation-associated fibroblasts markers, Wnt signaling pathway indicators, MHC-I markers, and MHC-II markers, we investigate more closely at the probable role of these fibroblasts (Fig. 6D). It was determined that MMP3, MMP11, and FAP—classic indicators of CAF—were substantially expressed in fibroblast clusters 2 and 5. Additionally, IL24, CCL2, CCL5, and chemokines with the C-X-C pattern were expressed at higher levels in fibroblast cluster 2 compared to other clusters. The above results confirmed that fibroblast cluster 2 and 5 were CAFs, which played a role in promoting ESCC progression in TME, and then we referred to them as CAF2 and CAF5. Also, we found that CAF2 up-regulated Wnt signaling pathway genes such WNT2 and WNT5A while down-regulating the expression of Wnt signaling inhibitor SFRP1, which was consistent with findings from the research on CAFs for gastric cancer [29]. MHC-I genes were considerably up-regulated in CAF2 and CAF5, whereas MHC-II genes were down-regulated, indicating a change in the antigen-presenting function of CAFs.

According to the pseudo-time analysis, fibroblast clusters 1, 3, and 6 were concentrated in the early stages of differentiation, while cluster 4 was present at every stage, and CAF2 and CAF5 were more evenly distributed in the middle and end stages (Supplementary Fig. 5C). It was found that CAF5 evolved into CAF2 in the middle stage of differentiation, and a tiny portion of it was differentiated from fibroblast clusters 1 and 4 when combined with the RNA rate diffusion diagram (Fig. 6E). It was also discovered that GSB, DCN, and CLU were highly expressed at the start of the development trajectory while PLAT, IGFBP2, MMP11, and WNT5A were highly expressed at the end of development, suggesting that they may be involved in the conversion of normal fibroblasts into CAFs and promote the progression of the tumor (Supplementary Figure 5D). The NC group had a higher concentration of fibroblasts in clusters 3 and 4, which differentiated in the direction of branch 2, while the CA group had a higher concentration of CAF2, which was concentrated in branches 1 and 2 (Fig. 6F). The chemo_CA group was more distinct from the immuno_CA group in the direction of branch 2.

We observed discrepancies between CAF2 and CAF5 in the TF analysis (Supplementary Fig. 5E). E2F8 and E2F7 dramatically decreased in CAF2 but significantly increased in CAF5, indicating that CAF5 had a greater capacity for proliferation than CAF2. In addition, we investigated the relationship between CAF and other cells and discovered that CAF2 controlled other fibroblast clusters via RARRES2-GPR1, CCL1-DPP4, and other pathways (Supplementary Fig. 5F). While CAF5 regulated other fibroblast clusters through the PDGFC-PDGFRA and WNT5A-ANTXR1 pathways, and influenced TAMs through the RARRES2-CMKLR1 pathway.

### Exploration of spatial distribution of immune cells in ESCC following neoadjuvant therapy

**Fig. 7:**
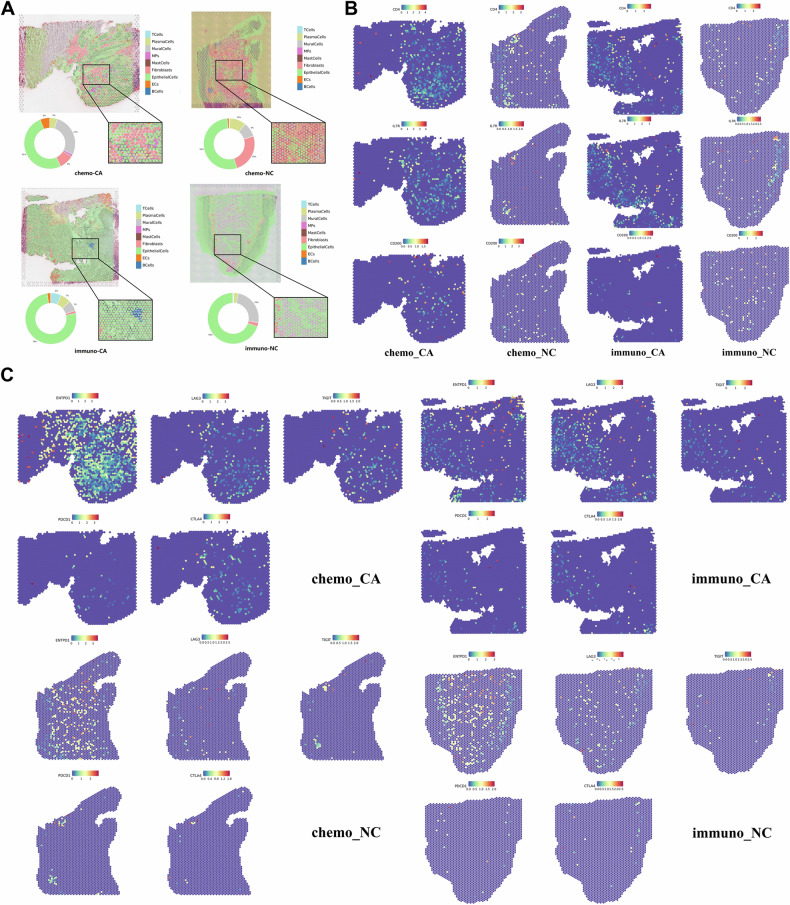
Spatial distribution of T cell subtypes and immune checkpoint markers in ESCC. **A** ST deconvolution pie of tissue samples. **B** Spatial distribution of T cell markers in four groups. **C** Spatial distribution of immune checkpoint markers in four groups. *P* < 0.05. *P < 0.05; **P < 0.01; ***P < 0.001.

The tissue sections of chemo_CA, chemo_NC, immuno_CA, and immuno_NC were chosen for ST analysis to more thoroughly define the transcriptome profile of ESCC and exhibit the spatial distribution information of TME. The division of the histopathologic regions was depicted in Supplementary Figure 6. Four tissue samples had a total of 2892, 2673, 3845, and 2954 spots, respectively, with a range of 1011 to 2084 genes being detected by each spot’s median number. Space Ranger software was used to carry out a quantitative and quality control study of gene expression (Supplementary Fig. 7A, B). The spot clustering findings were shown using the UMAP algorithm for dimensionality reduction analysis to retain more global structure data (Supplementary Fig. 7C). As seen in Supplementary Fig. 7D, assign the tissue sections to the spots clustering information using the spatial barcode. Cell types were identified at the spatial level using the deconvolution method (Fig. 7A). These samples’ cell type distribution matched that of the nine cell types identified by our scRNA-seq. Epithelial cells made up the majority of cell types in both groups. We essentially identified the primary cell types distributed in each part of the tissue sample by the expression of defining genes in spatial regions and the application of deconvolution. To analyze the relationship between each cell cluster and spatial region, the most thorough and understandable spatial distribution data was first acquired.

**Fig. 8:**
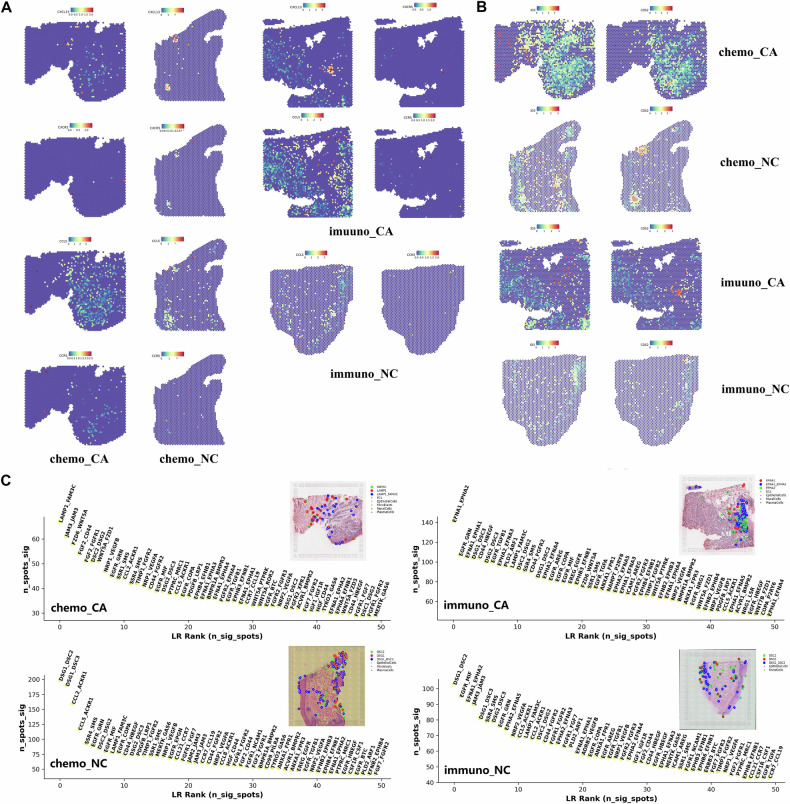
Spatial distribution of CXCL13/CXCR5, ID3 and CD52 in ESCC. **A** Spatial distribution of CXCL13-CXCR5 and CCL5-CCR5 in four groups. **B** Spatial distribution of ID3 and CD52 in four groups. **C** TOP50 interaction pairs in four groups. *P* < 0.05. *P < 0.05; **, *P* < 0.01; ***, *P* < 0.001.

In the beginning, we focused on the spatial distribution of some typical marker genes and immunological checkpoints. It appeared that T cells in the CA group had a more equal distribution throughout the tumor region, dispersed among the tumor cells, and had a greater expression level at the point where the tumor region and other regions met. T cells were more evenly distributed in the NC group at the intersections of several tissue regions and in T cell clusters that might be brought on by inflammation (Fig. 7B). In our investigation of the immune checkpoints’ spatial distribution, we found that ENTPD1 was extensively dispersed. It was sparsely distributed with a high expression level in the normal tissue region but densely distributed with a low expression level in the tumor region (Fig. 7C). The expression level increased with distance from the tumor region. The distribution of ENTPD1 was denser with a higher level in the immuno_CA group compared to the chemo_CA group. The fact that ENTPD1 is extensively expressed in vascular endothelial cells and fibroblasts in TME of ESCC in addition to immune cells like Treg, NK cells, and macrophages may account for its widespread dispersion. LAG3 and TIGIT were more widely dispersed in the tumor location, and their expression levels rose with increasing distance from the tumor. PDCD1 and CTLA4 were scattered sporadically in the tumor region with T cell aggregation, and they were more widely distributed in the CA group than the NC group. Similar to PDCD1 and CTLA4, which were scattered across the tumor region with evident T cell aggregation, CXCL13-CXCR5 had a similar spatial distribution. CXCL13 was much more prevalent than CXCR5 in all other groups (Fig. 8A), with the exception of the immuno_NC group, where their expression could not be seen. The expression level of CCL5-CCR5 was much higher than that of the tumor region, and it also exhibited T cell aggregation. CCL5-CCR5 was broadly dispersed in the tumor region and less so in the normal tissue region. Additionally, we investigated the study’s predictive indicators and looked at the spatial distribution traits of ID3 and CD52. It found that CD52 and ID3 were broadly dispersed throughout the four tissue samples, with a relatively low level of sparse distribution in the normal tissue region and a relatively high level of sparse distribution in the tumor region (Fig. 8B). Also, T cell focal aggregation showed significant CD52 expression.

For the purpose of precisely locating the various cell clusters in space and elucidating the spatial relationships between them, we performed scGSEA scores on ST samples using the DEGs acquired by scRNA-seq. In the tumor tissue region, we first observed that CD8 T cells were widely distributed and infiltrated (Supplementary Fig. 8A and 9A). ESCC is currently immune-hot tumor and is more responsive to immune checkpoint inhibitors when the results of the analysis discussed above are taken into account. It was found that the spatial distribution of CD4-C1-CCR7 overlate with that of macrophages, particularly in the chemo_CA group (Supplementary Fig. 8C). In the immuno_CA group, there was less spatial overlap between CD4-C1-CCR7 and macrophages (Supplementary Fig. 9A, C). It might be because the chemo_CA group has tightly packed plasma cells, which enhanced their spatial interaction. Interestingly, in both the chemo_CA group and the immuno_CA group, another naïve T cell cluster, CD4-C2-IL7R displayed a mutually exclusive condition with macrophages (Supplementary Fig. 8A and 9A).

We found that the spatial distribution of CD4-C3-FOXP3, NKT-C1-XCL, and DC in any group strongly overlapping. Furthermore, the regional distributions of CD4-C3-FOXP3 and Angio-Tams in the CA group were comparable, indicating that the HIF-1 signaling pathway may be employed to create a communication link between them. The chemo_CA group’s relationship between the two clusters was stronger than it was in the immuno_CA group, which suggested that immunotherapy might be a useful strategy for improving the hypoxic environment in TME. In the CA group, proMPs, LA-TAMs, CD4-C4-MKI67, and CD8-C2-MKI67 were all distributed very densely in space. The spatial distribution of these two exhausted T cell clusters was highly overlapping with that of CAF2 and CAF5 in the chemo_CA group, which were also obviously situated in the tumor location. However, in the immuno_CA group, CAF2 does not exhibit this spatial distribution, which may be explained by the immunotherapy’s reduction of the inflammatory response in the tumor locations. In the chemo_CA group, but not in the immuno_CA group, there was a strong correlation between the spatial distribution of RTM-TAMs and cDC1. A tendency toward tumor region dispersion was also present in fibroblast cluster 6, which was more pronounced in the chemo_CA group than in the immuno_CA group.

Finally, we explored the strongest interactions between the four samples, and Fig. 8C displays the top 50 rankings. LAMP3-FAM3C primarily affected plasma cells, fibroblasts, and epithelial cells along the tumor margin in the chemo_CA group. The immuno_CA group, which mostly worked on epithelial cells in normal epithelial regions bordering malignancies, contained the EFNA1-EPHA2 protein. The NC group had both DSC2-DSG1 and these molecules interacted in the epithelial cells of the normal epithelial area. According to a report, EGFR and HER2 can dimerize with EPHA2 to modify downstream signals and undergo malignant transformation [30]. This shows that EFNA1-EPHA2 in immuno_CA may contribute to malignant progression, including as the proliferation and metastasis of tumor cells, and that reducing its expression or blocking endogenous activation of EFNA1-EPHA2 regulatory axis may play a role in tumor inhibition.

Here, we report the TME of ESCC from the two dimensions of time and space using scRNA-seq and ST. The differences in immune cell types and components in TME under the two regimens were explored, and the underlying causes or mechanisms were investigated, through the comparative analysis of tumor tissues and matched adjacent normal tissues of patients receiving preoperative neoadjuvant chemotherapy and preoperative immunotherapy combined chemotherapy. To identify potential indicators for diagnosis and prognosis, it was also investigated how distinct cell clusters interacted with one another and were distributed spatially. This will establish the foundation for the future development and implementation of novel ESCC diagnosis and immunotherapy approaches.

## Discussion

The immune cell components in TME against tumor cells mainly involve effector T cells, NK cells, macrophages, DCs, which play killing roles through direct contact or indirect pathways [31–33]. In some cancers, tumor cells bind to PD-L1 ligands to inhibit the activation of CD8 T cells, a well-known immune escape mechanism. In addition, malignant progression occurs when CD8 T cells are in a state of exhaustion [34]. In our study, we identified two CD8 T cell clusters and four CD4 T cell clusters from T cells, which highly express cytokines IFNG, GZMB, NKG7, and checkpoint molecular genes LAG3, TIGIT, PDCD1, CLTA4, with obvious characteristics of exhausted T cells. Interestingly, some canonical cell cycle genes, such as CDKN3, CCNB2 and CCNB1, were highly expressed in the CD8-C2-MKI67. A study in 2020 reported as follows [35]: According to Ly108 and CD69, the development framework of was elaborated, and it was divided into four stages, namely, progenitor 1, progenitor 2, intermediate and terminal. Among them, the progenitor 2 stage is characterized by Ly108 + , which is in a proliferative state and has the ability to enter the blood, and the preferentially upregulated genes are enriched in the cell cycle, such as CDK1, CCNA2, and MKI67. It is suggested that CD8-C2-MKI67 is probably in the progenitor 2 stage. Furthermore, CD8-C2-MKI67 and CD4-C4-MKI67 both had very high proliferation scores, suggesting that exhaustion T cells was the main proliferating immune cell compartment in ESCC. Tregs are also an important part of the immune profile in TME, a key role in maintaining immune homeostasis. Inhibition of FOXP3+Tregs in TME can prevent CD8 T cells from effectively responding to tumor cells [36]. It was found that the proportion of CD4-C3-FOXP3 in the immuno-group decreased compared with the chemo-group, indicating that immunotherapy inhibited the proliferative function of Treg.

We used CD4 T data to select four genes, HSPH1, ATF3, NDUFB3 and HIST1H1E, to construct a risk score model for judging the prognosis, which may guide the clinical treatment of ESCC. Among these four genes, relatively more studies have been conducted on ATF3, a TFs member of the ATF/CREB family. It participates in cellular processes to adapt to extracellular and/or intracellular changes, and transduces signals from various receptors to activate or inhibit gene expression [37]. It was reported that ATF3 had prognostic significance as a novel tumor suppressor in ESCC, and it can inhibit ESCC through downregulation of ID1 [38, 39]. In our study, we found that compared with the NC group, the expression of ATF3 was up-regulated in the chemo_CA group, while down-regulated in the immuno_CA group, indicating that immunotherapy may inhibit the expression level of ATF3 through some unknown mechanism or pathway. In addition, we found that ESCC patients with high ATF3 expression had poor prognosis, and the expression level increased with the progression of ESCC stage, suggesting that ATF3 has the potential to serve as a biomarker. The role of other three CD4 T marker genes in ESCC needs to be solved urgently. Meanwhile, we also found two potential biomarkers in the immune profile, ID3 and CD52, which may assist in determining the prognosis of ESCC. ID3 has been found to be strongly associated with the anti-tumor effects of macrophages [40]. In addition, it can inhibit the exhaustion of anti-tumor CD8 T cells in liver cancer [41]. In short, ID3 has a regulatory role in a variety of cancers, acting as a bridge in the immune system [42, 43]. It was reported that the immune-related gene CD52 was a prognostic biomarker for breast cancer, melanoma [44, 45]. With further investigation, we believe that its predictive role in ESCC will be well reflected and applied in the clinic.

Two intriguing unusual clusters proMPs and mDCs were found in the MPs clusters. The primary proliferative elements in MPs should be ProMPs with significantly high expression of MKI67 and TOP2A, which accounted for a greater proportion in CA group. Mature mDCs specifically expressed CCR7, CCL17 and CCL22 to recruit infiltrating T cells, which transduced immune signals and recruits peripheral T cells in TME with strong migration and regulatory capabilities, thus playing an anti-tumor role. It may be a therapeutic target for ESCC in the future.

Nowadays, researchers have a deeper understanding of macrophages, whose types are not limited to M1 and M2 [46]. TAMs can restrict T cell function by producing immunosuppressive factors including TGFB and IL10. Recently, it was confirmed that high density of TAMs in ESCC was associated with shorter survival of patients, indicating that TAMs may become a prognostic biomarker for ESCC [47]. In this study, macrophages were subdivided into five subgroups. IFN-TAMs are highly expressed in CXCL10, ISG15, PD-L1, and M1 macrophage markers such as CD86 and MHC-II [48]. However, unlike M1-type macrophages, IFN-TAMs generally play an anti-tumor role and inhibit the immune response by degrading tryptophan and regulating Treg recruitment [49]. In this study, we hypothesized that IFN-TAMs in ESCC may serve as an organism protector against tumors. Angio-TAMs are generally enriched in the hypoxic region of the tumor immune microenvironment with high expression of angiogenesis markers [50]. Studies have reported that Angio-TAMS can promote tumor progression, and their abundance is associated with poor prognosis of patients, involving colorectal cancer, non-small cell lung cancer, melanoma [51, 52]. There is a common or stacked state of DEGs in these macrophage clusters, and there is a high possibility of mutual transformation among them.

It has been reported that CAFs can be used as a prognostic factor for ESCC patients receiving neoadjuvant therapy [53]. IL6 secreted by CAFs promotes the expression of ESCC cell receptor CXCR7 through the STAT3/NF-κB pathway, which is one of the reasons for ESCC cells resistance to cisplatin [54]. Here, we found that compared with the chemo_CA group, the proportion of CAFs in the immuno_CA group was slightly lower, which may be attributed to the inhibitory effect of Sintilimab on CAFs while activating the immune system to fight against tumor cells. Furthermore, the pseudo-time analysis presented that CAF2 not only differentiated from CAF5, but also a small number of normal fibroblasts could be transformed into CAF2. Genes with significantly increased expression in the middle and end stages of differentiation, such as MMP11, TNC and PLAT, may participate in this malignant transformation process.

ST provides an unbiased picture of the spatial composition, and many research teams have built valuable spatial profile based on it [55, 56]. It can also enhance the understanding of tumor substructure [57]. In this study, ST was used to analyze four tissue samples to spatially demonstrate the results obtained from the scRNA-seq analysis. It was found that the farther away from the tumor in TME, the higher the expression level of ENTPD1. ENTPD1 encodes CD39, which is an important extracellular nucleotidase [58]. CD39 is not only highly expressed in immune cells such as Treg, NK cells and macrophages, but also in vascular endothelial cells and fibroblasts, which may be one reason for its widespread expression in ESCC [59]. Among the strongest interactions in samples, we found that there were significant differences between the chemo_CA group and the immuno_CA group. The top three interactions in the former group were LAMP1-FAM3C, JAM3-JAM3 and FZD6-WNT5A, and in the latter group were EFNA1-EPHA2, EGFR-GRN, EFNA1-EPHA1. According to research reports, LAMP1, FAM3C and FZD6 all have the ability to predict the prognosis of ESCC, and WNT5A promotes ESCC metastasis by activating the HDAC7/SNAIL signaling pathway [60, 61]. EEFNA1 encodes EPH protein and is involved in regulating developmental events, while EPHA2 can interact with EPHA1 to regulate the movement and proliferation of tumor cells [62]. EPHA2 has also been confirmed to dimerize with EGFR and HER2, resulting in changes in downstream signals and malignant transformation [30]. This suggests that EFNA1-EPHA2-EPHA1, which strongly interact with each other in immuno_CA group, may cause malignant progression of tumor cells. Reducing their expression or blocking endogenous activation of the regulatory axis of EFNA1-EPHA2-EPHA may play a role in tumor inhibition [63]. EFNA1-EPHA2-EPHA1 may be an effective clinical therapeutic target or predictive biomarker for ESCC, and we will further study it in the future.

## Materials and methods

### Clinical sample collection

Six patients admitted to the esophageal oncology department of Tianjin Medical University Cancer Hospital, received neoadjuvant chemotherapy or neoadjuvant immunotherapy combined with chemotherapy. The former group was treated with nedaplatin or cisplatin combined with paclitaxel, while the latter group was treated with nedaplatin combined with paclitaxel and sindellizumab. These patients were newly treated and had not previously undergone any other surgery or treatment. The tumors in the gastroesophageal junction were excluded, and only the cancers in the esophagus were retained. The pathological type was esophageal squamous cell carcinoma (ESCC), excluding adenocarcinoma and other tumor types. Histological grade was moderately or poorly differentiation. The period of radical surgery was September 2021 until February 2022. Informed consent papers were signed by patients or family members. Tianjin Medical University Cancer Hospital’s ethics committee gave its approval for this study (E2020169). The pathology division of Tianjin Medical University Cancer Hospital provided the tissue samples for IHC paraffin-embedded analysis. From December 2019 through June 2022, individuals who underwent radical surgery could be followed up.

### Quality control, dimensionality reduction, and cluster analysis

Cells were screened if they had 200–5000 genes and a UMI count of less than 30000, and cells with a mitochondrial content of more than 20% were eliminated. A total of 88951 cells were left after low-quality cells were eliminated for further examination. Seuratv3.1.2 was used for dimensionality reduction and clustering, and NormalizeData and ScaleData were used to normalize and scale all gene expression. The first 2000 variable genes are chosen for principal components analysis (PCA) by FindVariableFeautres. Cluster the cells using the first 20 major components from Findclusters. After that, the UMAP method was used to display the cells in two dimensions.

### Analysis of differentially expressed genes (DEGs)

The DEGs analysis was done on nine significant cell clusters. The Wilcox likelihood ratio test for the default parameters served as the foundation for the study, which was carried out with FindMarker in Seurat v3.1.2. DEGs selection criteria: the mean Log(FC) was greater than 0.25 and the percentage of cells expressing in clusters was greater than 10%. The heat map/point map/violin map was then visually presented.

### Cell cluster annotation

Each cell cluster was located using a combination of data from the literature and manual annotation of the expression of canonical marker genes discovered in DEGs. To display each cell clusters of the marker gene expression, Seurat v3.1.2 DoHeatmap DotPlot/Vlnplot map was used.

### Copy number variations (CNV) analysis

The InferCNV package was used to detect CNV in malignant cells. To examine the intensity changes in gene expression at each place on the tumor genome, immune cells were employed as the baseline reference. A gene’s sequence was determined by where it was on each chromosome. Selection criteria for genes: More than 20 cells were expressed. The residual normalized expression values served as the reference point for the relative expression values, which had a 1.5 standard deviation upper bound. Uphyloplot2 can be used to show the final visualization, which uses the sliding window size of 101 genes to smooth out the relative expression on each chromosome and eliminate any potential impacts of gene-specific expression.

### Evaluation of cell cycle effects

Since each stage of the cell cycle has important marker genes and is regulated by cell cycle-related proteins, it is possible to score each cell’s cycle gene set using CellCycleScoring in the Seurat package in order to determine how the cell affects the cycle effect and then to visualize the histogram/UMAP map.

### Transcriptional regulatory network analysis of pySCENIC

The investigation of the transcriptional regulatory network was performed using pySCENIC, and the analysis of the scRNA-seq data allowed for the inference of the relevant transcription factors (TFs) and the gene regulatory network. The probable TF-target regulatory relationship was identified based on gene co-expression analysis, and cis-regulatory motif analysis was carried out for each co-expression module to maintain direct targets with the proper upstream regulators and significant enrichment. AUC evaluated the regulatory factors’ target genes, determined the activity of the regulators, categorized the cells based on their activity, and then displayed the cells using heat maps/particular scatter plots.

### Cell interaction analysis

Using CellphoneDB v2.1.0, cell cluster was used as the object of the investigation of cell interactions. To assess the cell connection between the two various cell types, the average amount of each receptor’s expression in each cell cluster was computed based on the known ligands and their receptors. By randomly rearranging the cell type label, calculating the average expression of the interactions 1000 times, obtaining the distribution, and comparing the average expression to the determined average expression of the identified cell type, displacement tests were used to determine the *P*-value of a pair of ligand-receptor (LR) interactions. The criteria of significant cell interaction: *P* < 0.05. Finally, the visualization was carried out through Cytoscape.

### Pseudo-time trajectory analysis

By sequencing cells in accordance with their progression and reconstructing their trajectory as they differentiate or go through a biological transformation, pseudo-temporal trajectory analysis using Monocle2 demonstrated the differentiation or transformation process of cell clusters. Seurat v3.1.2 FindVairableFeatures was used to choose the first 1000 highly variable genes from cell clusters to reduce the dimensionality using DDRTree. Plot_cell_trajectory was used to illustrate the trajectory visually.

### RNA rate analysis

Using the BAM file including cell clusters and their reference genomes, velocyto, and scVelo in Python, RNA rate analysis was carried out. The rate of gene splicing was assessed by comparing the relative abundance of newly formed unspliced mRNA with fully formed spliced mRNA. The rate and direction of changes in the transcriptome during the dynamic process should be revealed by consistent signals in the scRNA-seq data. The cluster analysis performed by Seurat 3.1.2 was then projected to UMAP for visual display.

### Spatial cell type tag score

ssGSEA scoring algorithm was used to score subcell type characteristics to explore the spatial distribution of subpopulations, and the scoring algorithm was robust to ST data. Using the top 20 upregulated cell type-specific genes (excluding mitochondrial and ribosomal genes) from the scRNA-seq data, feature gene sets were generated. Finally, Seurat’s SpatialFeaturePlot was used to depict feature scores. Cell-cell interactions in tissue sample space were studied using the spatial ligand-receptor interaction stLearn v 0.4.7 to pick out important areas for LR interactions from candidate LR in the Cell Phone EDB database. For further examination, any LR with a score lower than 20 spots was excluded. Benjamini-Hochberg corrected the *P*-value modification. Finally, the built-in visual function of stLearn was used to implement the visual presentation of LR.

### Statistical analysis

Data analysis was done statistically using GraphPad Prism 8.0. The two groups were analyzed using the *T*-test, such as chemo-group and immuno-group, low group and high group, etc. Multiple groups were compared using one-way ANOVA, such as chemo NC/CA group and immuno NC/CA group, etc. The data were shown using mean ± standard deviation (M ± SD), and correlation analysis was performed using the Spearman method. The statistical significance of the difference between the data is shown by *P* < 0.05. *P < 0.05; **P < 0.01; ***P < 0.001.

## Supplementary information

Supplementary Figure Legends, Supplementary Materials and methods

SUPPLEMENTAL MATERIAL 1

SUPPLEMENTAL MATERIAL 2

SUPPLEMENTAL MATERIAL 3

SUPPLEMENTAL MATERIAL 4

SUPPLEMENTAL MATERIAL 5

SUPPLEMENTAL MATERIAL 6

SUPPLEMENTAL MATERIAL 7

SUPPLEMENTAL MATERIAL 8

SUPPLEMENTAL MATERIAL 9

## Data Availability

The datasets generated during and analysed during the current study are available from the corresponding author on reasonable request.
