# Supplementary material for: A spatiotemporal comparative analysis on tumor immune microenvironment characteristics between neoadjuvant chemotherapy and preoperative immunotherapy for ESCC
Source: Cell Death Dis. 2024 Sep 10;15(9):663. doi: 10.1038/s41419-024-06986-y (PMC11387609; doi:10.1038/s41419-024-06986-y)
Supplement: Supplementary file 1 — Supplementary Figure Legends, Supplementary Materials and methods [file 41419_2024_6986_MOESM1_ESM.pdf]

Supplementary Materials and Methods

Construction of risk scoring model

The CD4 T cell marker genes were used to constructed the risk scoring model based on the TCGA database. $P < 0.01$ was regarded as significant for univariate analysis, which was utilized to screen CD4 T cell marker genes related with ESCC prognosis. The risk coefficient (Coef) of each gene was determined after screening the genes affecting prognosis using LASSO regression analysis. The T cell marker gene expression (Expr) and its related regression coefficient (Coef) were used to construct the risk score: risk score $=\sum_{n}^{i}(coef \times expr)$. Based on median risk scores, ESCC patients were separated into high-risk and low-risk groups, overall survival was evaluated, and receiver operating characteristic (ROC) curves were used to determine how well the risk score model predicted outcomes.

Spatial RNA-seq data processing, integration, and visualization

Spatial RNA-seq data from 10X Visium capture regions (each containing 5000 barcode points) were processed using the Space Ranger analysis software, and the resulting matrix files were analyzed using the Seurat v4.0.1 software package. In the normalization process, each spot feature count was divided by the total number of cells, multiplied by the scale factor (1e6), and Log1p was used for logarithmic conversion. Use FindVariableFeatures to select the first 2000 high-variable genes from the Seurat object. These genes were PCA with RunPCA, the data dimension was reduced to the first 20 principal components, then scaled and centered in the dataset, which can be visualized on UMAP with RunUMAP. Meanwhile, FindNeighbors and Findclusters were run together to cluster points on the PCA space using the SNN algorithm to generate an initial cluster. Use FeaturePlot and SpatialFeaturePlot to visualize marker genes. Based on the results of the Seurat analysis, stLearn absorbed spatial distance, tissue morphology and gene expression from the raw counts of the data and images. The initial step was to filter out genes that were only present in one cell and normalize and Log1p. Then, stLearn extracted advanced features from the tiled images, performed PCA analysis on the gene expression data, and applied stSME to normalize the log-converted data. Finally, on the stSME normalized data, several

clusters were clustered using Louvain method. To estimate spatial cell cluster composition, spatial transcriptome deconvolution was performed with Seurat label transformation. Data labeled cell types from the scRNA-seq and Spatial RNAseq data were re-normalized and PCA by Seurat using SCTransform and RunPCA. FindTransferAnchors found an anchor that integrates with two data sets. The basic composition of cell types was predicted by the TransferData method to deconvolve each spatial voxel, resulting in a table containing the proportion of cell types for each spot in the spatial data, which was passed to stLearn for deconvolution visualization and quantification of cell type composition in each cluster.

Supplementary Figure Legends

Supplementary Figure 1. Single cell transcriptome map and major cell types in ESCC tumor microenvironment.

(A) Proportion of each cell type in the total sample. (B) UMAP of epithelial cells. (C) Expression of typical marker genes in each cluster of epithelial cells. (D) CNV analysis of immune cells and malignant cells. Red represents amplification, blue represents deletion. (E) UMAP of cell cycle of epithelial cells and cell cycle ratio of four clusters. $P < 0.05$. *， $P < 0.05$； **， $P < 0.01$； ***， $P < 0.001$

Supplementary Figure 2. Clustering and subtype analysis of T cells in ESCC tumors after neoadjuvant chemotherapy or preoperative immunotherapy

(A) KEGG functional enrichment analysis bubble map of T cell clusters. (B) Expression levels of CTLA4 and PDCD1 in T cell clusters. (C) Proportion of T cell clusters in all samples. (D) Developmental trajectory of T cell clusters. (E) Dynamic changes of T cell gene expression. (F) Heat map of TFs of T cell clusters in four groups. $P < 0.05$. *， $P < 0.05$； **， $P < 0.01$； ***， $P < 0.001$

Supplementary Figure 3. ID3 and CD52 serve as markers to predict the efficacy of two neoadjuvant treatment regimens for ESCC

(A) Heat map of ID3, CD52 and immune cells in malignant tumor. (B) Scatter plot of correlation between ID3, CD52 and multiple immune cells in ESCC. $P < 0.05$. *， $P <$

0.05；**，$P < 0.01$；***，$P < 0.001$

Supplementary Figure 4. Clustering and subtype analysis of MPs

(A) UMAP of macrophage clusters and all tissue samples. (B) Violin diagram of CCR7, CCL17, and CCL22 expression levels in DCs. (C) The proportion of MPs clusters in all samples. (D) Developmental trajectory of MPs clusters. (E) Dynamic changes of MPs gene expression. (F) M1/M2 functional feature score box of macrophages clusters. $P < 0.05$. *，$P < 0.05$；**，$P < 0.01$；***，$P < 0.001$

Supplementary Figure 5. Analysis of fibroblast clusters and CAF in ESCC after two neoadjuvant treatment regimens

(A) The proportional contribution of four groups to each fibroblast clusters. (B) Proportion of fibroblast clusters in four groups. (C) Developmental trajectory of fibroblast clusters. (D) Dynamic changes of fibroblast gene expression. (E) Heat map of TFs of fibroblast clusters in four groups. (F) A bubble map of CAFs interacting with other cells. $P < 0.05$. *，$P < 0.05$；**，$P < 0.01$；***，$P < 0.001$

Supplementary Figure 6. Exploration of spatial distribution of immune cells in ESCC following neoadjuvant therapy

Pathological region division of four tissue samples. Different colors represent different tissue areas. $P < 0.05$. *，$P < 0.05$；**，$P < 0.01$；***，$P < 0.001$

Supplementary Figure.7 Exploration of spatial distribution of immune cells in ESCC following neoadjuvant therapy

(A) Violin diagram of sample quality control of ST spots. (B) UMAP of sample quality control of ST spots. (C) UMAP of ST spot dimension reduction clustering. (D) In situ diagram of tissue sections of spot clusters. $P < 0.05$. *, $P < 0.05$; **, $P < 0.01$；***，$P < 0.001$

Supplementary Figure.8 Exploration of spatial distribution of immune cells in ESCC following neoadjuvant therapy

(A) scGSEA spatial score of T cells in chemo_CA group. (B) scGSEA spatial score of fibroblasts in chemo_CA group. (C) scGSEA spatial score of MPs in chemo_CA group. $P < 0.05$. *，$P < 0.05$；**，$P < 0.01$；***，$P < 0.001$

Supplementary Figure.9 Exploration of spatial distribution of immune cells in

ESCC following neoadjuvant therapy

(A) scGSEA spatial score of T cells in immuno_CA group. (B) scGSEA spatial score of fibroblasts in immuno_CA group. (C) scGSEA spatial score of MPs in immuno_CA group. $P < 0.05$. *，$P < 0.05$；**，$P < 0.01$；***，$P < 0.001$
