# Supplementary figures and images for: A spatiotemporal comparative analysis on tumor immune microenvironment characteristics between neoadjuvant chemotherapy and preoperative immunotherapy for ESCC
Source: Cell Death Dis. 2024 Sep 10;15(9):663. doi: 10.1038/s41419-024-06986-y (PMC11387609; doi:10.1038/s41419-024-06986-y)

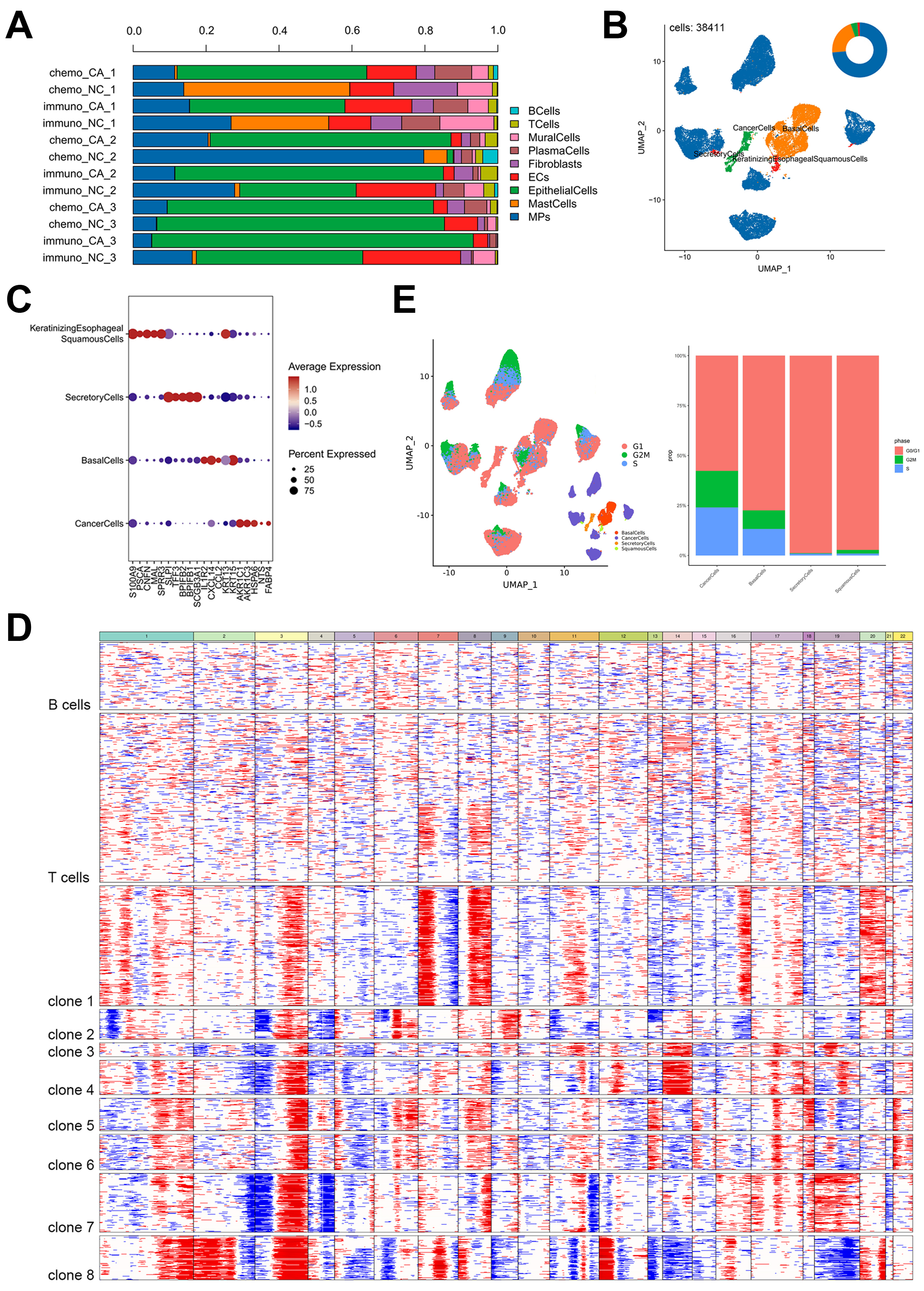

Supplement: Supplementary file 2 — SUPPLEMENTAL MATERIAL 1 [file 41419_2024_6986_MOESM2_ESM.jpg]

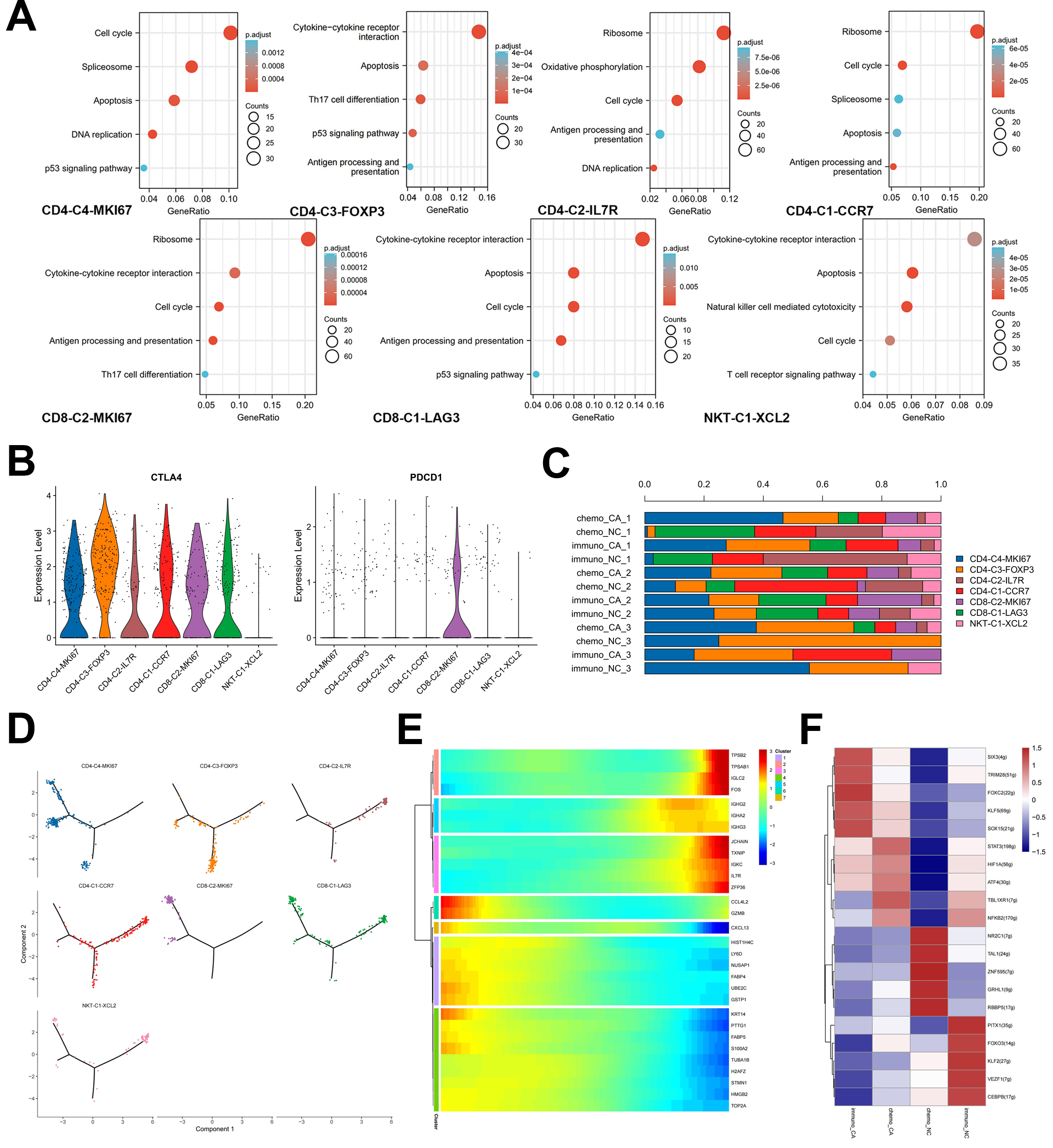

Supplement: Supplementary file 3 — SUPPLEMENTAL MATERIAL 2 [file 41419_2024_6986_MOESM3_ESM.jpg]

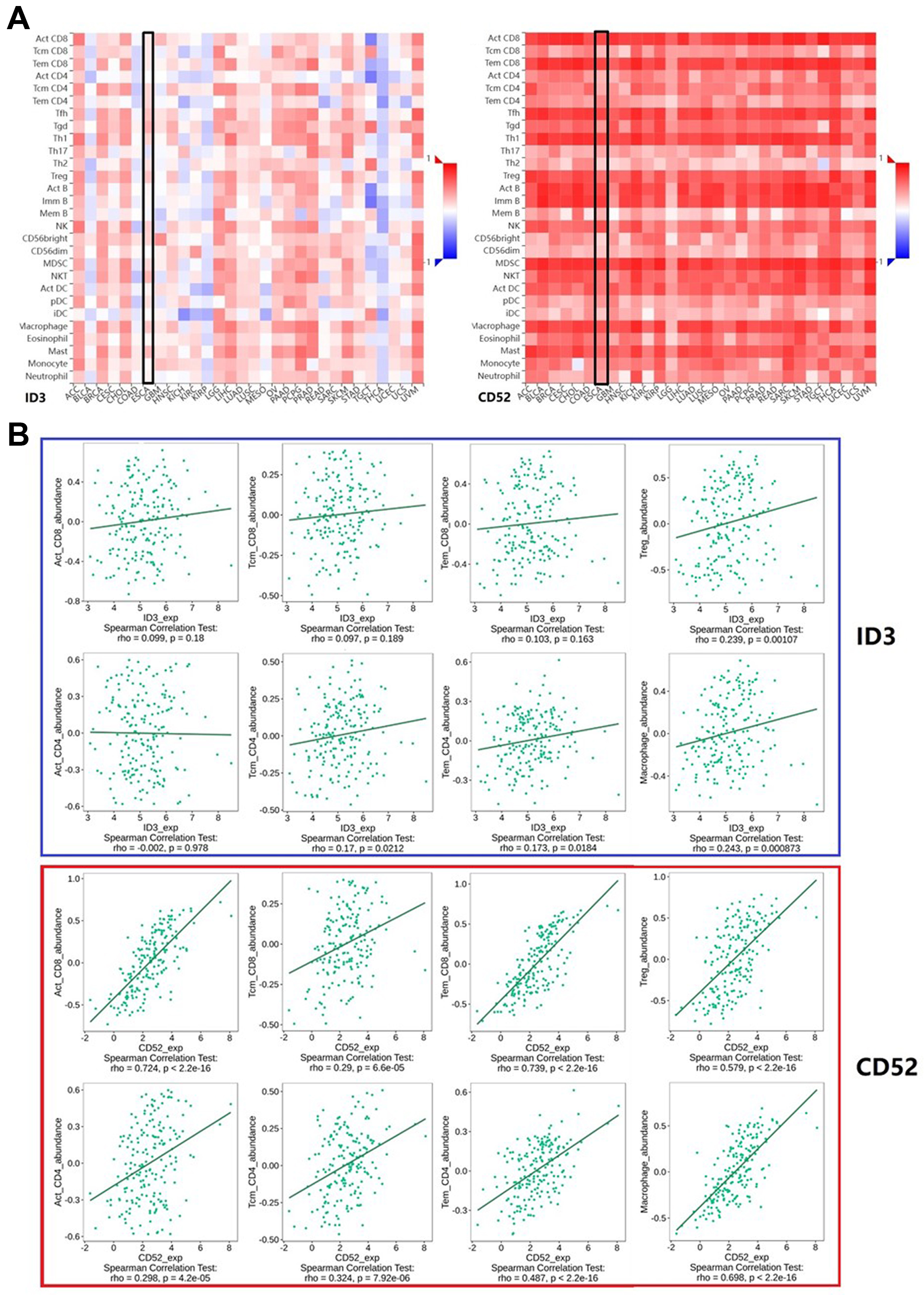

Supplement: Supplementary file 4 — SUPPLEMENTAL MATERIAL 3 [file 41419_2024_6986_MOESM4_ESM.jpg]

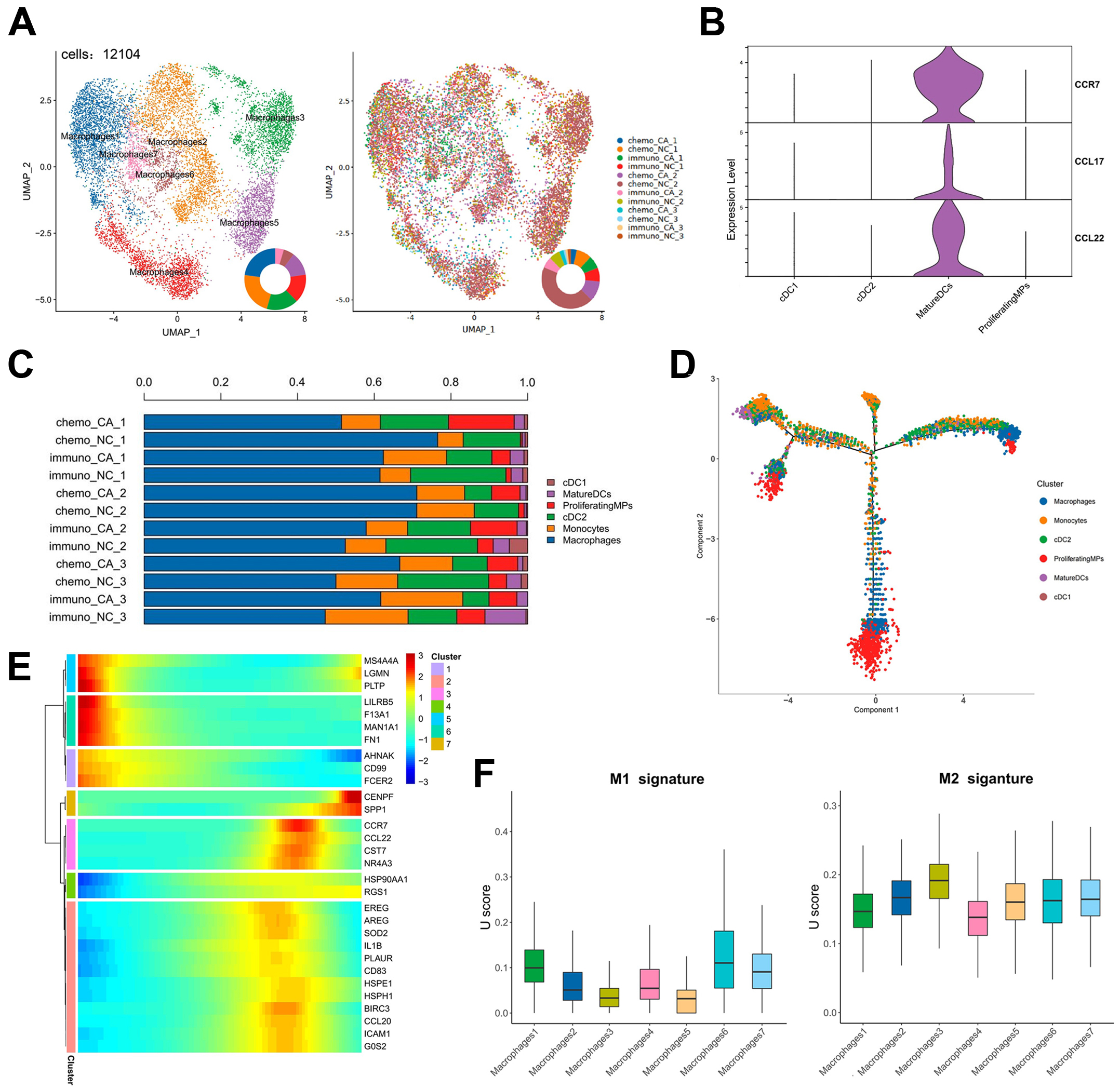

Supplement: Supplementary file 5 — SUPPLEMENTAL MATERIAL 4 [file 41419_2024_6986_MOESM5_ESM.jpg]

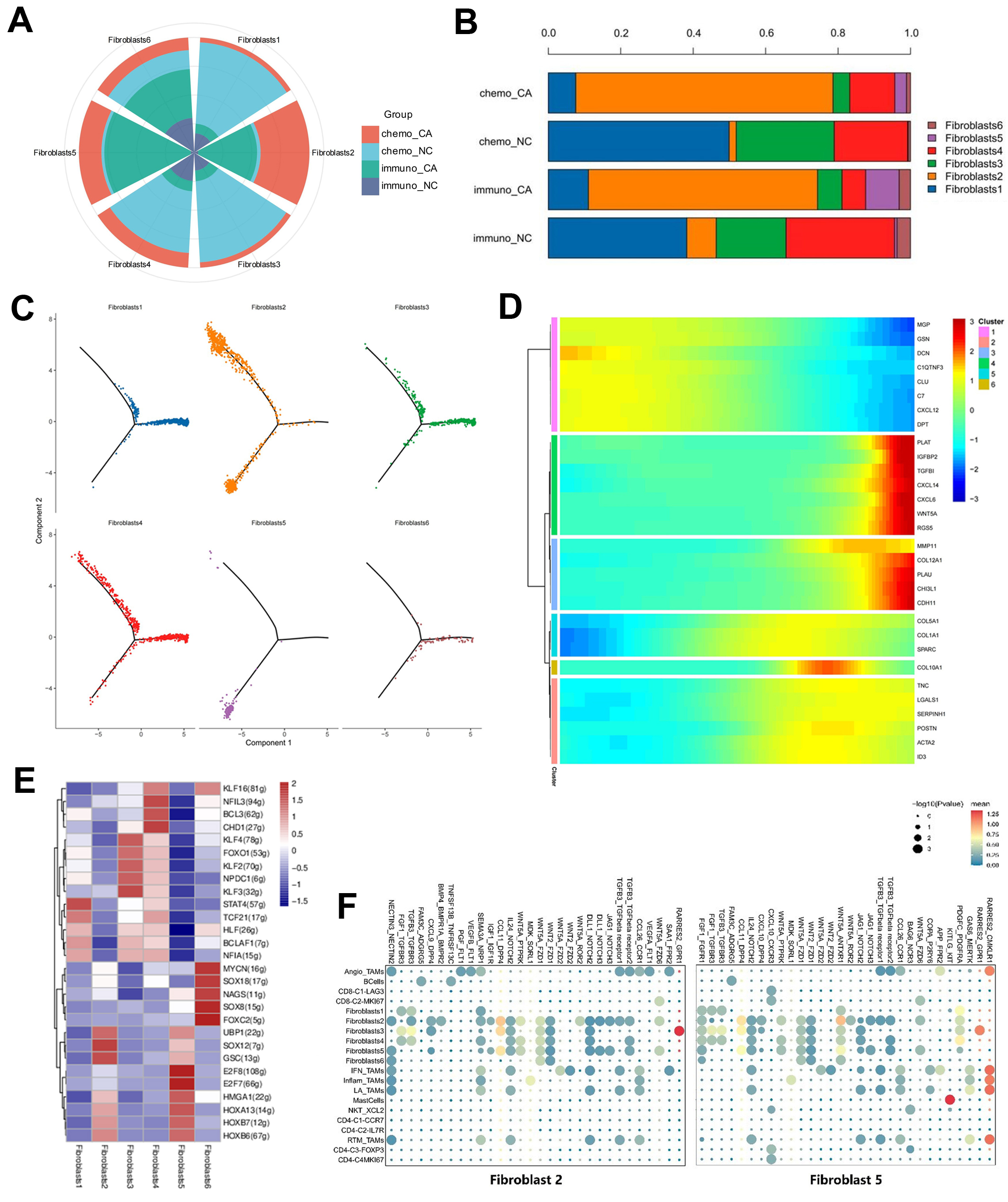

Supplement: Supplementary file 6 — SUPPLEMENTAL MATERIAL 5 [file 41419_2024_6986_MOESM6_ESM.jpg]

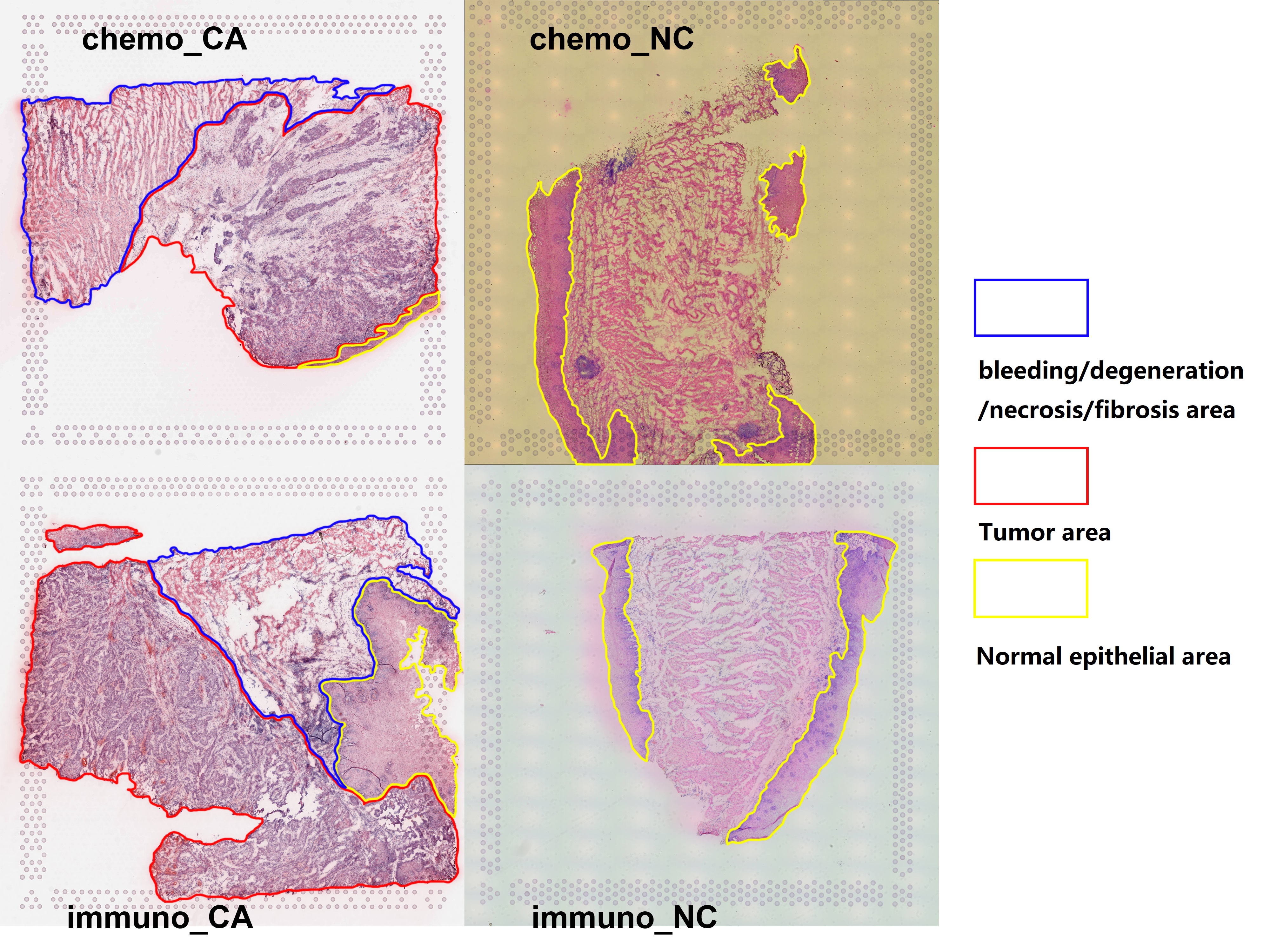

Supplement: Supplementary file 7 — SUPPLEMENTAL MATERIAL 6 [file 41419_2024_6986_MOESM7_ESM.jpg]

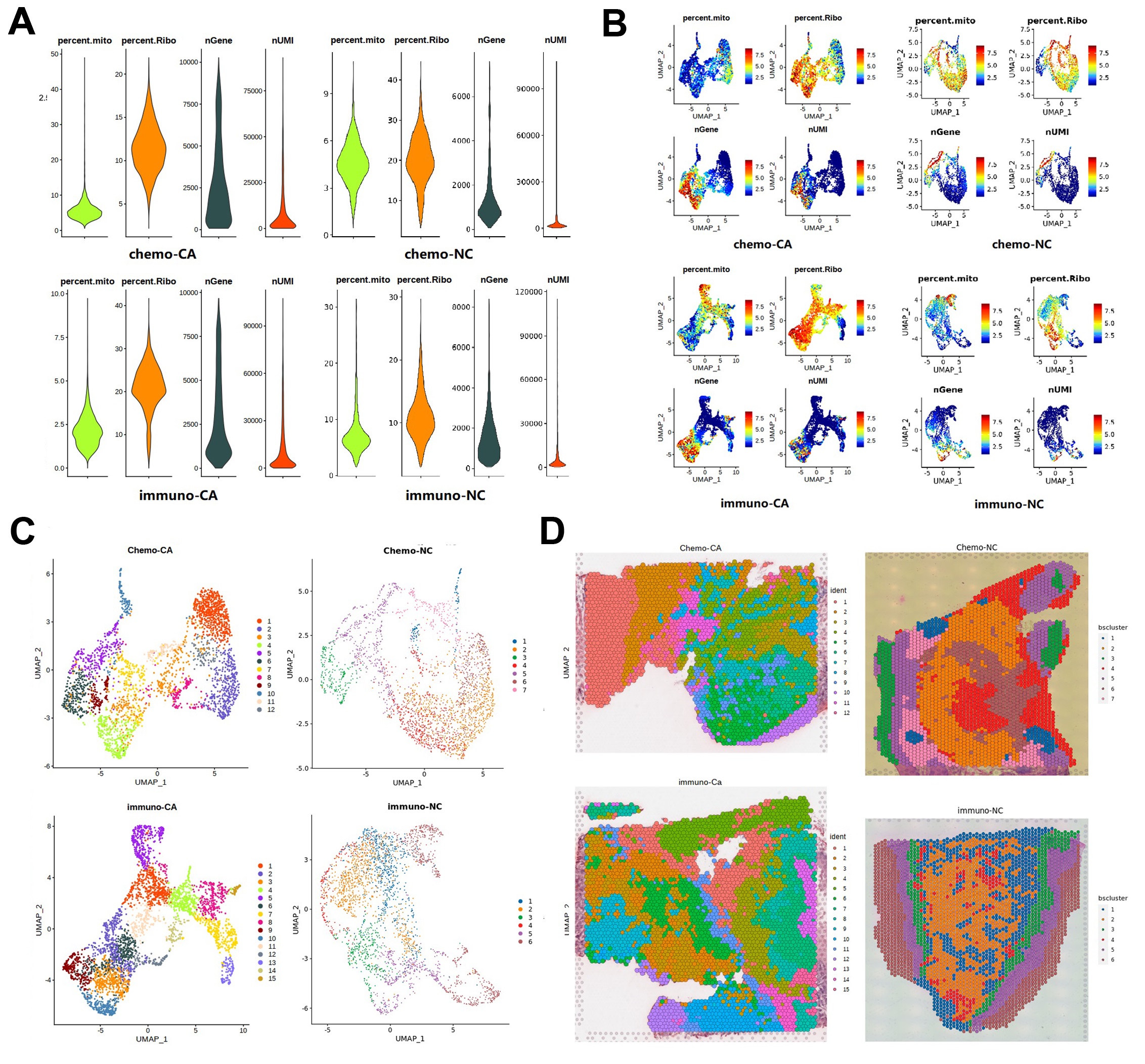

Supplement: Supplementary file 8 — SUPPLEMENTAL MATERIAL 7 [file 41419_2024_6986_MOESM8_ESM.jpg]

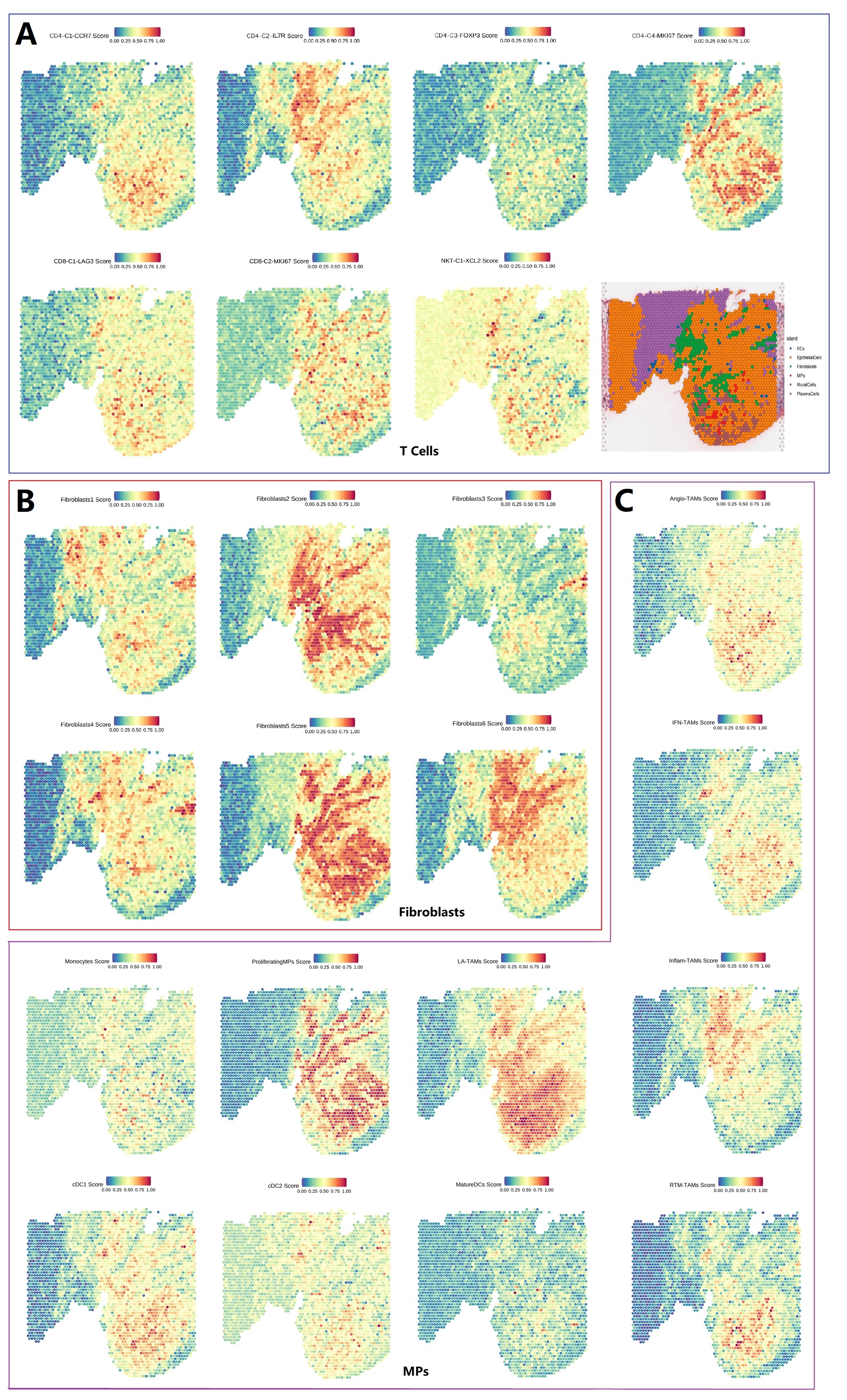

Supplement: Supplementary file 9 — SUPPLEMENTAL MATERIAL 8 [file 41419_2024_6986_MOESM9_ESM.jpg]

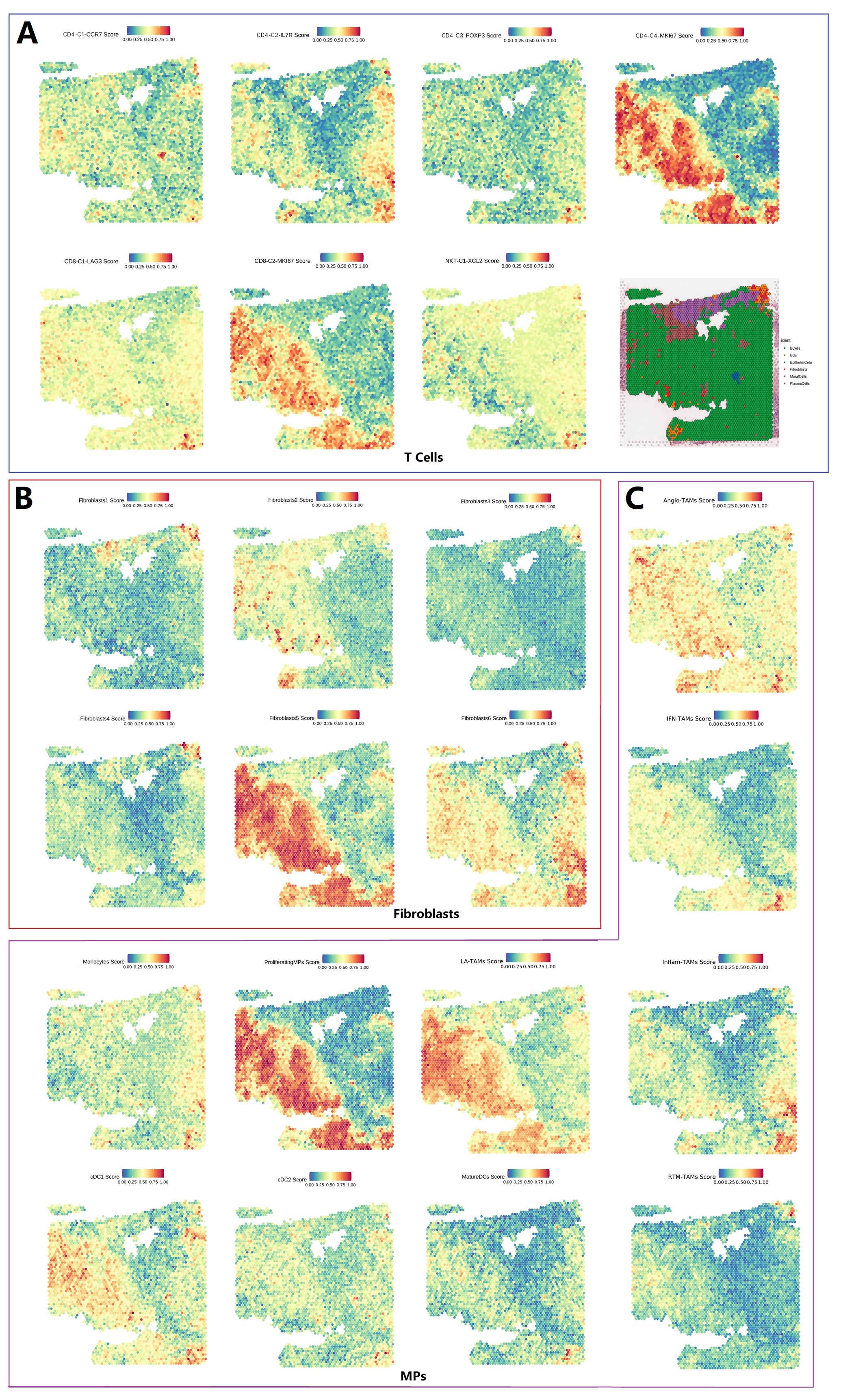

Supplement: Supplementary file 10 — SUPPLEMENTAL MATERIAL 9 [file 41419_2024_6986_MOESM10_ESM.jpg]
